# An integrated modelling approach for targeted degradation: insights on optimization, data requirements and PKPD predictions from semi- or fully-mechanistic models and exact steady state solutions

**DOI:** 10.1007/s10928-023-09857-9

**Published:** 2023-04-29

**Authors:** Sofia Guzzetti, Pablo Morentin Gutierrez

**Affiliations:** DMPK, Research and Early Development, Oncology R&D, AstraZeneca, Cambridge, UK

**Keywords:** Protein degradation, PROTACs, Mechanistic modelling, Binding kinetics, Degradation kinetics, Global sensitivity analysis, Model identifiability

## Abstract

The value of an integrated mathematical modelling approach for protein degraders which combines the benefits of traditional turnover models and fully mechanistic models is presented. Firstly, we show how exact solutions of the mechanistic models of monovalent and bivalent degraders can provide insight on the role of each system parameter in driving the pharmacological response. We show how on/off binding rates and degradation rates are related to potency and maximal effect of monovalent degraders, and how such relationship can be used to suggest a compound optimization strategy. Even convoluted exact steady state solutions for bivalent degraders provide insight on the type of observations required to ensure the predictive capacity of a mechanistic approach. Specifically for PROTACs, the structure of the exact steady state solution suggests that the total remaining target at steady state, which is easily accessible experimentally, is insufficient to reconstruct the state of the whole system at equilibrium and observations on different species (such as binary/ternary complexes) are necessary. Secondly, global sensitivity analysis of fully mechanistic models for PROTACs suggests that both target and ligase baselines (actually, their ratio) are the major sources of variability in the response of non-cooperative systems, which speaks to the importance of characterizing their distribution in the target patient population. Finally, we propose a pragmatic modelling approach which incorporates the insights generated with fully mechanistic models into simpler turnover models to improve their predictive ability, hence enabling acceleration of drug discovery programs and increased probability of success in the clinic.

## Introduction

Mechanistic modelling has proven extremely valuable not only in enhancing the understanding of both traditional and new modalities mechanism of action [1–4], but also in supporting and guiding compound optimization by shedding light on Structure-Activity Relationship (SAR). Especially for emerging new modalities where little is known about the mechanism or where technology lags behind science in generating reliable data on biological or pharmacological quantities of interest, inexpensive high-throughput model simulations can to some extent “bridge the gap” between science and technology by predicting the response of a biological system in scenarios of interest and, more importantly, by identifying which mechanistic parameters are key drivers of the response. Such quantitative understanding can ultimately provide a robust rationale to identify which missing data would be most informative in building an understanding of the pharmacology, and hence which technologies need prioritization for data generation.

On the other hand, though, the use of mechanistic models to explain available data can be impractical and potentially even misleading without a thorough understanding of the *amount* and *type* of data that is required to *uniquely* identify the model parameters. While modelling software is designed to always return parameter estimates, if the model structure is excessively articulated (over-parametrized) for the data, or, conversely, if the amount and/or type of data is insufficient or too simplistic to inform the building blocks of a mechanistic model, multiple parameter estimates can provide an equally optimal data fit. However, the *uncertainty* on those estimates is typically high, or their reliability low, i.e. the output values are highly unlikely to be truly representative of the biological, chemical or pharmacological quantity they encode (e.g., protein baselines, endogenous turnover rates, on/off binding rates, drug-induced degradation rates, ...). In such case the risk is over-interpreting the parameter values, potentially leading to wrong decisions or scientific conclusions at different levels in a drug discovery cascade, from compound optimization to assessment of therapeutic potential.

Although much has been published on identifiability analysis of differential equations to address this issue [5, 6], the minimal required data on target kinetics can be challenging to generate, and even when this is not the case its generation requires long timelines and significant resources. Actually, in many cases kinetics data provide a level of detail that goes beyond the necessary and sufficient needs for pragmatically understanding the underlying mechanism of action (MoA). On the contrary, the steady state (i.e., dynamical equilibrium) of a biological or pharmacological system can be enough to provide insights on the MoA and hence to build a robust and reliable predictive model from higher throughput and more easily accessible data [7–10]. Moreover, the steady state can often be mechanistically described by simpler (non-linear) algebraic equations directly derived from a parent set of differential equations, for which an *exact* solution might even be available (depending on the mathematical structure of the system) [7, 8, 11, 12]. Although the resulting mathematical formulae may still retain some level of complexity (and they typically do), they can facilitate the identification of independent surrogate parameters, i.e. compositions of the original parameters into sums, ratios or other functional forms, which can lead to a reduction of the model parametrization and, ultimately, to improved model reliability. Such approach to identifiability can further be combined with global sensitivity analysis [13] to identify which of the original mechanistic parameters dominate the system response variability, and hence whose experimental measurements would be most impactful.

Whenever an exact steady state solution cannot be calculated or its complexity is still prohibitive, semi-mechanistic models such as indirect-response (turnover) models [14] offer an alternative option. Such models are easier to use in practice due to the smaller number of parameters that require fitting, however while they can successfully explain the data in specific studies or experimental settings, they lack information on binding kinetics or baseline levels, which makes them prone to failure at predicting the response for different compounds or cell lines [15, 16].

In this manuscript we propose an integrated modelling approach which leverages the benefits of each type of model to mitigate the limitations of the others. Firstly we show how *exact* mechanistic steady state solutions of the MoA of binary- and ternary-complex degraders can (i) provide noise-free insight on the role of each system parameter in driving the pharmacological response regardless of its specific value, (ii) suggest which mechanistic knowledge can be confidently extracted from single time point data and (iii) which additional data needs to be collected to enhance the mechanistic understanding of the system, and (iv) ultimately, inform compound optimization, data generation and resource prioritization.

Secondly, we show how global sensitivity analysis of fully mechanistic models can help to identify the key drivers of the response.

Finally, we propose a pragmatic modelling approach which incorporates the insights generated with fully mechanistic models into simpler turnover models to improve their predictive ability, hence enabling acceleration of drug discovery programs and increased probability of success in the clinic.

## Methods

### Exact solution of bilinear systems

The life cycle of biological entities and their interaction with chemical compounds can be described via non-linear systems of ordinary differential equations (ODEs), where the type of non-linearity is often bilinear or at most quadratic [17]. Exact steady-state solutions of such systems are challenging to compute and rarely available in closed form, due to potentially many chemical species involved and corresponding interactions, resulting in a large number of non-linear equations. Nevertheless, in some cases implicit relationships can be obtained and solved numerically. Not only, they can inform on the system identifiability, i.e. which parameters (or surrogates thereof) can be uniquely estimated from the data. A mathematical method to obtain an implicit, exact steady state solution to chemical reaction networks with bilinear rate laws is described in [18] and is summarized in Appendix A. Briefly, such method applies thoughtful algebraic manipulations to leverage the linear component of the system while segregating the non-linear part to its core, which can then be solved numerically.

### Monovalent degraders

In this paper we refer to compounds that induce degradation of a protein of interest (PoI) by binding solely to it as Monovalent Degraders (MDs). It is very likely that other components are required for degradation of the protein (e.g. the proteasome), nevertheless no assumption is made here on the mechanism of degradation. The only assumption is that the compound needs to bind solely to the PoI to induce its degradation with no requirement to bind any other component of the system (differently from bivalent degraders).

#### A mechanistic model for monovalent degraders

**Fig. 1:**
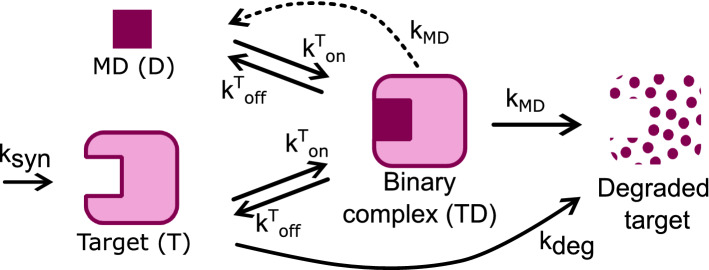
A mechanistic model for monovalent degraders

We assume that under endogenous conditions (i.e. in absence of compound) the target protein synthesis and degradation are zero- and first-order processes, respectively, with corresponding rates $k_{\text {syn}}$, $k_{\text {deg}}$. When compound is added, binding kinetics governed by on/off rates $k^{\text {T}}_{\text {on}}$, $k^{\text {T}}_{\text {off}}$ leads to the formation of a binary complex, which induces degradation at a first order rate $k_{\text {MD}}$. Upon degradation the compound is released and recycled (Fig. 1).

Such mechanism can be mathematically described by the following system of ODEs:

1 $$\left\{ {\begin{array}{*{20}l} {\frac{{d{\text{T}}}}{{dt}} = k_{{{\text{syn}}}} - k_{{{\text{deg}}}} \cdot {\text{T}} + k_{{{\text{off}}}}^{{\text{T}}} \cdot {\text{TD}} - k_{{{\text{on}}}}^{{\text{T}}} \cdot {\text{T}} \cdot {\text{D}}} \hfill \\ {\frac{{d{\text{D}}}}{{dt}} = - k_{{{\text{on}}}}^{{\text{T}}} \cdot {\text{T}} \cdot {\text{D}} + (k_{{{\text{off}}}}^{{\text{T}}} + k_{{{\text{MD}}}} ) \cdot {\text{TD}}} \hfill \\ {\frac{{d{\text{TD}}}}{{dt}} = k_{{{\text{on}}}}^{{\text{T}}} \cdot {\text{T}} \cdot {\text{D}} - (k_{{{\text{off}}}}^{{\text{T}}} + k_{{{\text{MD}}}} ) \cdot {\text{TD}}} \hfill \\ \end{array} } \right.$$

with initial conditions $\text {T}(0)=\text {T}_0=k_{\text {syn}}/k_{\text {deg}}$, $\text {D}(0)=\text {D}_0$, $\text {TD}(0)=0$, where $\text {T}$, $\text {D}$, and $\text {TD}$ represent target, drug, and binary complex time-varying concentrations, respectively. Being the MD recycled, the *total* MD concentration $\text {D}_0= \text {D}+\text {TD}$ is preserved. As a result, the last (or second) equation is redundant as the binary complex (or MD) concentration at any time can be obtained via the linear conservation law as

2 $$\begin{aligned} \text {TD}(t) = \text {D}_0- \text {D}(t). \end{aligned}$$

Since we are interested in the steady state, we set the derivatives in (1) to 0 to obtain a steady state model:

3 $$\left\{ {\begin{array}{*{20}l} {k_{{{\text{syn}}}} - k_{{{\text{deg}}}} \cdot {\text{T}} + k_{{{\text{off}}}}^{{\text{T}}} \cdot {\text{TD}} - k_{{{\text{on}}}}^{{\text{T}}} \cdot {\text{T}} \cdot {\text{D}} = 0} \hfill \\ { - k_{{{\text{on}}}}^{{\text{T}}} \cdot {\text{T}} \cdot {\text{D}} + (k_{{{\text{off}}}}^{{\text{T}}} + k_{{{\text{MD}}}} ) \cdot {\text{TD}} = 0} \hfill \\ {{\text{TD}} + {\text{D}} = {\text{D}}_{0} .} \hfill \\ \end{array} } \right.$$

Note that, while in system (1) $\text {T}$, $\text {D}$ and $\text {TD}$ are time-dependent variables, they are constant in (3) by definition of steady state (the same notation has been used for the sake of simplicity). Because system (3) is non-linear, calculating an exact solution is not straightforward. Nevertheless, this type of non-linearity (bilinear) falls within the category of tractable systems which can be solved with the mathematical method developed in [18].

### PROTACs

**Fig. 2:**
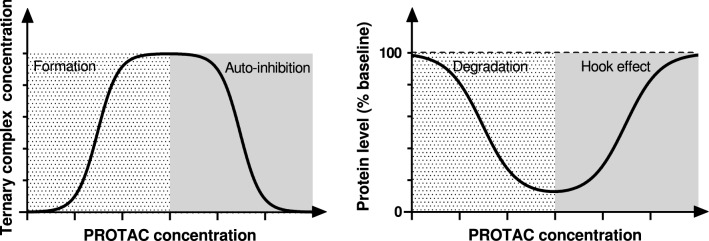
Ternary complex levels as a function of PROTAC concentration in a pure binding system (left); target levels relative to baseline as a function of PROTAC concentration in a biological setting (right)

Proteolysis targeting chimeras (PROTACs) are a novel drug modality that fosters degradation catalysis by co-opting an E3 ligase (e.g. Cereblon or VHL) to tag the targeted PoI for turnover by the proteasome. Such mechanism of action heavily relies on the formation of a ternary complex with the PoI and E3 ligase, which is known to be liable to auto-inhibition, i.e., impairment of ternary complex formation at high PROTAC concentrations due to an excess of PoI- or E3 ligase-PROTAC binary complex [19, 20]. In other words, while in a two-body binding system the amount of binary complex increases monotonically with the binder concentration up to ligand saturation following a sigmoidal relationship, ternary complex formation can decrease as the PROTAC concentration increases beyond a critical threshold and is hence typically described by a *double* sigmoidal (bell-shaped) function (Fig. 2, left). From a pharmacological perspective, such binding dynamics in a biological setting can result into the so-called hook effect [19], i.e., a loss of degradation at high PROTAC concentrations, with the implication that maximal effect can only be achieved within a certain concentration window, whose center and breadth are highly specific to each molecule (Fig. 2, right). It is worth noting that not all PROTACs display auto-inhibition in practice. Efforts to reduce the hook effect whenever present have been summarized by Cecchini et al. [21], nevertheless it is fair to say that additional work is required to fully understand how to pragmatically minimize this phenomenon.

#### PROTACs mechanistic modelling

**Fig. 3:**
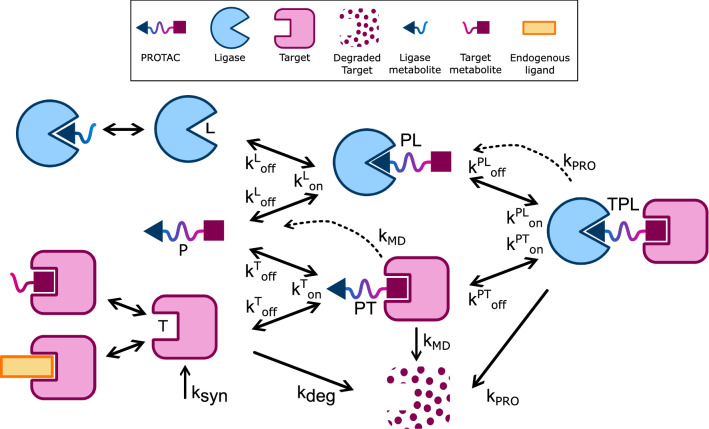
A PROTAC mechanistic model

Ternary complex formation (and subsequent degradation) can happen through two different pathways: from a PROTAC-target ($\text {PT}$) or PROTAC-ligase ($\text {PL}$) binary complex, where the extent of the contribution of each pathway is dictated by binding affinities, PROTAC concentration, and target and E3 ligase baselines (Fig. 3). Once the ternary complex is formed the PoI is degraded while PROTAC and E3 ligase are released and recycled back into the system. It is important to keep in mind that PROTACs are not themselves degraders, rather degradation catalysts. Therefore, the apparent “PROTAC degradation rate” ($k_{\text {PRO}}$) is in fact a surrogate, composite parameter that synthesizes (i) ubiquitin transfer rate (which depends on the stereochemistry), (ii) ubiquitination rate and (iii) proteasomal degradation rate as a whole. While the stereochemistry can be optimized – at least in principle – to facilitate the ubiquitin transfer, the latter two parameters are endogenous to the biological system and can dictate the overall degradation kinetics (i.e. can be rate-limiting). This underscores the importance of understanding biological differences across cell lines, which cannot be conceived separately from the compound’s kinetics.

In vitro data for different targets suggests that three degradation scenarios can occur to the PoI bound into a $\text {PT}$ binary complex:

1. Endogenous degradation (flat line around the baseline)
2. Low-moderate degradation (more efficient than endogenous degradation, less efficient than ternary complex degradation), for example when the target warhead is a MD itself (shallow degradation curve)
3. Stabilization, i.e., the compound inhibits the endogenous degradation machinery (sigmoidal curve above baseline).

Such observation justified the introduction of a binary complex degradation rate ($k_{\text {MD}}$) as an independent model parameter (equal to, greater or less than the endogenous degradation rate, respectively, in the three scenarios described above).

This basic mechanistic model can be further customized to include, e.g., competition with metabolites or endogenous ligands (Fig. 3) – which may be relevant for early PROTACs whose PoI ligand consists of a pre-existing small molecule inhibitor which binds to an active site of the target protein [22], or by unfolding the PROTAC degradation rate in a series of transit compartments to better describe the ubiquitination or de-ubiquitination process, as done, e.g., in [23, 24]. Since the relevance of these model components may be target or chemo-type specific, for the sake of generality they will not be included in this analysis, although the methodology utilized here can be applied to an extended version of the model as well (provided the assumption of total PROTAC and total ligase conservation is met).

The governing equations are derived from mass balancing principles and they read as follows:

4 $$\left\{ {\begin{array}{*{20}l} {\frac{{d{\text{T}}}}{{dt}} = k_{{{\text{syn}}}} - k_{{{\text{deg}}}} \cdot {\text{T}} + k_{{{\text{off}}}}^{{{\text{PL}}}} \cdot {\text{TPL}} + k_{{{\text{off}}}}^{{\text{T}}} \cdot {\text{PT}} - k_{{{\text{on}}}}^{{\text{T}}} \cdot {\text{T}} \cdot {\text{P}} - k_{{{\text{on}}}}^{{{\text{PL}}}} \cdot {\text{PL}} \cdot {\text{T}}} \hfill \\ {\frac{{d{\text{PL}}}}{{dt}} = k_{{{\text{off}}}}^{{{\text{PL}}}} \cdot {\text{TPL}} + k_{{{\text{PRO}}}} \cdot {\text{TPL}} - k_{{{\text{off}}}}^{{\text{L}}} \cdot {\text{PL}} + k_{{{\text{on}}}}^{{\text{L}}} \cdot {\text{L}} \cdot {\text{P}} - k_{{{\text{on}}}}^{{{\text{PL}}}} \cdot {\text{PL}} \cdot {\text{T}}} \hfill \\ {\frac{{d{\text{PT}}}}{{dt}} = k_{{{\text{off}}}}^{{{\text{PT}}}} \cdot {\text{TPL}} - k_{{{\text{MD}}}} \cdot {\text{PT}} - k_{{{\text{off}}}}^{{\text{T}}} \cdot {\text{PT}} + k_{{{\text{on}}}}^{{\text{T}}} \cdot {\text{T}} \cdot {\text{P}} - k_{{{\text{on}}}}^{{{\text{PT}}}} \cdot {\text{PT}} \cdot {\text{L}}} \hfill \\ {\frac{{d{\text{TPL}}}}{{dt}} = - k_{{{\text{PRO}}}} \cdot {\text{TPL}} - k_{{{\text{off}}}}^{{{\text{PL}}}} \cdot {\text{TPL}} - k_{{{\text{off}}}}^{{{\text{PT}}}} \cdot {\text{TPL}} + k_{{{\text{on}}}}^{{{\text{PL}}}} \cdot {\text{PL}} \cdot {\text{T}} + k_{{{\text{on}}}}^{{{\text{PT}}}} \cdot {\text{PT}} \cdot {\text{L}}} \hfill \\ {\frac{{d{\text{L}}}}{{dt}} = - k_{{{\text{on}}}}^{{\text{L}}} \cdot {\text{L}} \cdot {\text{P}} - k_{{{\text{on}}}}^{{{\text{PT}}}} \cdot {\text{PT}} \cdot {\text{L}} + k_{{{\text{off}}}}^{{\text{L}}} \cdot {\text{PL}} + k_{{{\text{off}}}}^{{{\text{PT}}}} \cdot {\text{TPL}}} \hfill \\ {\frac{{d{\text{P}}}}{{dt}} = - k_{{{\text{on}}}}^{{\text{T}}} \cdot {\text{T}} \cdot {\text{P}} - k_{{{\text{on}}}}^{{\text{L}}} \cdot {\text{L}} \cdot {\text{P}} + k_{{{\text{MD}}}} \cdot {\text{PT}} + k_{{{\text{off}}}}^{{\text{T}}} \cdot {\text{PT}} + k_{{{\text{off}}}}^{{\text{L}}} \cdot {\text{PL}}} \hfill \\ \end{array} } \right.$$

with initial conditions $\text {T}(0)=\text {T}_0$, $\text {L}(0)=\text {L}_0$, $\text {P}(0)=\text {P}_0$, $\text {PL}(0)=\text {PT}(0)=\text {TPL}(0)=0$, where $\text {T}$, $\text {L}$, $\text {P}$, $\text {PT}$, $\text {PL}$, $\text {TPL}$ stand for target, ligase, PROTAC, PROTAC-target complex, PROTAC-ligase complex, and ternary complex concentration, respectively, and will be referred to as *states* of the system (dependence on time *t* has been suppressed to ease the notation). Model parameters are defined in Table 1. Note that on/off rates are correlated via cooperativity $\alpha$ as [25]:

5 $$\begin{aligned} \dfrac{k^{\text {PT}}_{\text {on}}}{k^{\text {PT}}_{\text {off}}}= \alpha \cdot \dfrac{k^{\text {T}}_{\text {on}}}{k^{\text {T}}_{\text {off}}}, \qquad \dfrac{k^{\text {PL}}_{\text {on}}}{k^{\text {PL}}_{\text {off}}}= \alpha \cdot \dfrac{k^{\text {L}}_{\text {on}}}{k^{\text {L}}_{\text {off}}}, \end{aligned}$$

and since the proportionality constant $\alpha$ is the same in (5), the relationship between on/off rates can be synthetically expressed as

6 $$\begin{aligned} \dfrac{k^{\text {T}}_{\text {off}}}{k^{\text {T}}_{\text {on}}}\dfrac{k^{\text {PT}}_{\text {on}}}{k^{\text {PT}}_{\text {off}}}= \dfrac{k^{\text {L}}_{\text {off}}}{k^{\text {L}}_{\text {on}}}\dfrac{k^{\text {PL}}_{\text {on}}}{k^{\text {PL}}_{\text {off}}}. \end{aligned}$$

This means that, even though the binding kinetics is governed by 8 parameters, only 7 of them are independent. In other words, knowing any 7 on/off rates is sufficient to calculate the remaining one via Eq (6). Adding endogenous synthesis and degradation rates ($k_{\text {syn}}$, $k_{\text {deg}}$) as well as binary and ternary complex degradation rates ($k_{\text {MD}}$, $k_{\text {PRO}}$) gives a total of 11 independent parameters.

**Table 1:** PROTAC model parameters: nomenclature and description

Parameter	Units	Rate	Reaction
$k_{\text {syn}}$	$\mu M\ h^{-1}$	Endogenous synthesis	$\emptyset \rightarrow \text {T}$
$k_{\text {deg}}$	$h^{-1}$	Endogenous degradation	$\text {T}\rightarrow \emptyset$
$k_{\text {MD}}$	$h^{-1}$	Binary cpx degradation	$\text {PT}\rightarrow \emptyset + \text {P}$
$k_{\text {PRO}}$	$h^{-1}$	Ternary cpx degradation	$\text {TPL}\rightarrow \emptyset + \text {PL}$
$k^{\text {T}}_{\text {on}}$	$\mu M^{-1}h^{-1}$	On	$\text {T}+ \text {P}\rightarrow \text {PT}$
$k^{\text {T}}_{\text {off}}$	$h^{-1}$	Off	$\text {PT}\rightarrow \text {T}+ \text {P}$
$k^{\text {L}}_{\text {on}}$	$\mu M^{-1}h^{-1}$	On	$\text {L}+ \text {P}\rightarrow \text {PL}$
$k^{\text {L}}_{\text {off}}$	$h^{-1}$	Off	$\text {PL}\rightarrow \text {L}+ \text {P}$
$k^{\text {PL}}_{\text {on}}$	$\mu M^{-1} h^{-1}$	On	$\text {T}+\text {PL}\rightarrow \text {TPL}$
$k^{\text {PL}}_{\text {off}}$	$h^{-1}$	Off	$\text {TPL}\rightarrow \text {T}+\text {PL}$
$k^{\text {PT}}_{\text {on}}$	$\mu M^{-1} h^{-1}$	On	$\text {L}+\text {PT}\rightarrow \text {TPL}$
$k^{\text {PT}}_{\text {off}}$	$h^{-1}$	Off	$\text {TPL}\rightarrow \text {L}+\text {PT}$

Under the assumption of total PROTAC and total E3 ligase conservation the differential equations for free PROTAC and free E3 ligase are redundant as they can be obtained from conservation laws as

7 $$\left\{ {\begin{array}{*{20}l} {{\text{L}}(t)\, = \,{\text{L}}_{0} \, - \,{\text{PL}}(t)\, - \,{\text{TPL}}(t)} \hfill \\ {{\text{P}}(t)\, = \,{\text{P}}_{0} \, - \,{\text{PT}}(t)\, - \,{\text{PL}}(t)\, - \,{\text{TPL}}(t),} \hfill \\ \end{array} } \right.$$

where $\text {L}_0$ and $\text {P}_0$ are the ligase baseline and PROTAC concentration, respectively.

The steady state of the system is obtained by setting each derivative in (4) to 0, and the same method described in [18] previously adopted for MDs can be applied here.

### Global sensitivity analysis

Sensitivity analysis is a powerful tool to understand how and to what extent each model parameter (input) affects the response (output) [13]. *Local* sensitivity analysis studies the system states variability as a single parameter varies, all the others being fixed. While this approach is extremely convenient for its simplicity of implementation and easiness of interpretation, it can be misleading as the sensitivity of the response to one parameter can depend on the values of all the other fixed parameters. In other words, the model output can be sensitive to a parameter $p^\star$ for a given set of the other parameter values and at the same time insensitive to $p^\star$ for a different set of fixed values. Therefore, this approach can be useful and unbiased only if confidence in the fixed parameters is high.

Differently, *global* sensitivity analysis studies the output variability over the whole parameter space, i.e. as *all* model parameters are changed *simultaneously*. As a result, the response variability characterization is more robust as it only depends on the assumed or observed *distribution* of each parameter on a given feasible *range* rather than on a single value [26]. At the same time, though, visualizing the response variability and quantifying the contribution of each parameter to it is increasingly challenging with the dimension of the parameter space: it is well known that the number of points to accurately sample a parameter space grows exponentially with the dimension of the parameter space itself (“curse of dimensionality”). In other words, if *N* samples are sufficient to describe the distribution of a single parameter, an order of $N^P$ points will be required to equivalently accurately sample *P* parameters. For instance, if 10 samples are used for each parameter of the PROTAC model (4) the total number of required samples would be approximately 100 *billions* ($10^{11}$). Each set of the 100 billion parameter combinations will generate a model output, and visualizing and interpreting 100 billion model simulations is clearly more challenging than plotting 10 of them (as a single parameter changes), as well as more computationally expensive.

A plethora of mathematical tools to quantify the impact of each parameter on the response and to tackle the curse of dimensionality are available. In this work Sobol indices [27] are used to assess the fraction of total variability associated with each parameter, which is a random variable represented via Polynomial Chaos Expansions (PCEs) [28–31]. Parameters are assumed to be uniformly distributed around a given mean and standard deviation, and are hence accordingly represented by first-order PCE of Legendre polynomials, which maximize the convergence rate according to the Askey scheme [32, 33]. Parameter uncertainty is propagated in the system via Non-Intrusive Spectral Projection (NISP) [34]. As a result, the stochastic model output can be represented as a PCE as well, whose coefficients can be easily used to calculate Sobol indices. In order to reduce the computational cost of sampling associated with NISP without compromising accuracy, Smolyak sparse quadratures are employed [35]. The sparsity level has been manually increased until no significant change in the Sobol indices estimates was observed. Uncertainty propagation via NISP and Sobol indices calculation was handled with the C++ library UQTk developed at the Sandia National Laboratories [36], embedded in a MATLAB implementation of the mechanistic PROTAC model (4).

### Experimental data and modelling

#### In vitro

Test compounds were evaluated at 12 concentrations obtained with a 1:3 dilution factor, with two replicates per concentration. Remaining PoI levels were measured via Western Blots, ERD9 [37], Immunofluorescence [38] or HiBit technology [39] and expressed as a fraction of protein in DMSO treated cells (i.e. baseline).

The following bi-sigmoidal model describing the remaining fraction of target at steady state ($\widehat{\text {T}}_{\text {SS}}$) as a function of concentration (*C*) was fitted to each individual dose-response at steady state to capture any potential hook effect and calculate maximal degradation and potency:

8 $$\begin{aligned} \widehat{\text {T}}_{\text {SS}}(C) = 1 - \text {E}_{max}\dfrac{C^{h}}{C^{h}+\text {EC}_{50}^{h}}\left( 1 - \dfrac{\text {E}_{loss}}{\text {E}_{max}}\dfrac{C}{C+\text {IC}_{50}} \right) . \end{aligned}$$

Eq (8) describes the response as a combination of two sigmoidal curves with half-maximal concentrations $\text {EC}_{50}$ and $\text {IC}_{50}$, respectively. $\text {E}_{max}$ represents the overall maximal effect, while $\text {E}_{loss}$ can be interpreted as the fraction of degradation lost to the hook effect. To avoid over-parametrization the Hill coefficient of the sigmoid corresponding to the hook effect was fixed to 1, while it was estimated (*h*) for the sigmoid corresponding to increasing degradation. Because the response is the result of the contribution of two distinct sigmoidal curves, the observed potency ($\text {DC}_{50}$) may not exactly correspond to $\text {EC}_{50}$, therefore it was calculated numerically from (8) as the concentration delivering half-maximal effect. The concentration corresponding to maximal degradation ($\text {DC}_{max}$) was also calculated numerically as the root of the first derivative, and maximal degradation as $\text {D}_{max}= 1 -\widehat{\text {T}}_{\text {SS}}(\text {DC}_{max})$. Note that in absence of the hook effect ($\text {E}_{loss}=0$) Eq. (8) reduces to a simple sigmoidal function where $\text {E}_{max}= \text {D}_{max}$ and $\text {EC}_{50}= \text {DC}_{50}$.

Whenever dose-response time courses were available, model (8) was embedded into the following turnover model describing the fraction of target at any given time ($\widehat{\text {T}}$):

9 $$\begin{aligned} \dfrac{d\widehat{\text {T}}}{dt} = k_{\text {deg}}\cdot \left( 1 - \dfrac{\widehat{\text {T}}}{\widehat{\text {T}}_{\text {SS}}(C)} \right) , \end{aligned}$$

where the endogenous fractional turnover rate $k_{\text {deg}}$ was estimated or fixed to experimental data obtained from Stable Isotope Labeling with Amino acids in Cell culture (SILAC) [40].

Experimental data in Sect. 4.1.3 has been generated with AstraZeneca proprietary Selective Estrogen Receptor Degraders (SERDs) [41–45].

Endogenous PoI or E3 ligase levels in different cell lines were assessed via Western Blots.

#### In vivo

In vivo data was generated in NSG mice implanted with a patient-derived xenograft tumor model. C-PROTAC-006 was dosed orally on a daily schedule for three days at 30 mg/kg, 60 mg/kg or 100 mg/kg. Plasma concentration at different time points was quantified via Liquid Chromatography with tandem Mass Spectrometry (LC-MS/MS). Remaining protein levels relative to vehicle baseline levels were assessed by Western Blots at 6h, 24h, 48h after the last dose.

A one-compartmental pharmacokinetic model with first-order absorption was fitted to the plasma concentration data and used as a driver of the pharmacodynamic model (9) parametrized from *in vitro* data generated in the same cell line to obtain predictions of *in vivo* degradation kinetics.

## Results

### Monovalent degraders

#### Exact steady state solution

By applying the method in [18] the following expression can be obtained, which implicitly describes the free target at steady state (see Appendix B.1):

10 $$\begin{aligned} \dfrac{k_{\text {syn}}}{k_{\text {MD}}}\dfrac{k^{\text {T}}_{\text {off}}+k_{\text {MD}}}{k^{\text {T}}_{\text {on}}}\cdot \dfrac{1}{\text {T}} - \dfrac{k_{\text {deg}}}{k_{\text {MD}}}\cdot \text {T}+ \dfrac{k_{\text {syn}}}{k_{\text {MD}}} - \dfrac{k_{\text {deg}}}{k_{\text {MD}}} \dfrac{k^{\text {T}}_{\text {off}}+k_{\text {MD}}}{k^{\text {T}}_{\text {on}}}= \text {D}_0. \end{aligned}$$

Since *total* protein amounts ($\text {T}+ \text {TD}$) *relative* to baseline are more easily accessible experimentally, through some manipulations an expression for the fraction of total remaining target can also be obtained as a function of the free MD concentration (see Appendix B.2):

11 $$\begin{aligned} \dfrac{\text {T}_{tot}}{\text {T}_0}(\text {D}) = \dfrac{k_{\text {deg}}}{k_{\text {MD}}} +\left( 1 - \dfrac{k_{\text {deg}}}{k_{\text {MD}}}\right) \cdot \dfrac{\dfrac{k_{\text {deg}}}{k_{\text {MD}}}\dfrac{k^{\text {T}}_{\text {off}}+k_{\text {MD}}}{k^{\text {T}}_{\text {on}}}}{\text {D}+ \dfrac{k_{\text {deg}}}{k_{\text {MD}}} \dfrac{k^{\text {T}}_{\text {off}}+k_{\text {MD}}}{k^{\text {T}}_{\text {on}}}}. \end{aligned}$$

Notice how Eq. (11) has the structure of a decreasing sigmoidal curve of the form

12 $$\begin{aligned} \dfrac{\text {T}_{tot}}{\text {T}_0}(\text {D}) = \widehat{\text {T}}_{min}+\left( 1 - \widehat{\text {T}}_{min}\right) \cdot \dfrac{\text {DC}_{50}}{\text {D}+ \text {DC}_{50}}, \end{aligned}$$

with top plateau and Hill coefficient equal to 1, and bottom plateau ($\widehat{\text {T}}_{min}$), maximal degradation ($\widehat{D}_{max}$) and concentration for half-maximal degradation ($\text {DC}_{50}$) respectively equal to

13 $$\begin{aligned} \widehat{\text {T}}_{min}= \dfrac{k_{\text {deg}}}{k_{\text {MD}}}, \quad \widehat{D}_{max}= 1 - \widehat{\text {T}}_{min}, \quad \text {DC}_{50}= \left( 1-\widehat{D}_{max}\right) \cdot \dfrac{k^{\text {T}}_{\text {off}}+k_{\text {MD}}}{k^{\text {T}}_{\text {on}}}, \end{aligned}$$

which is consistent with [46].

### PROTACs

#### Exact steady state solution

**Fig. 4:**
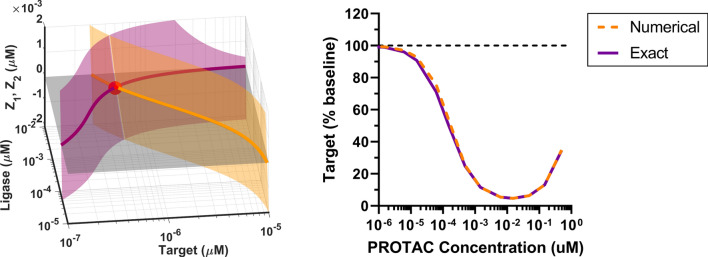
Left: Example of a solution to the PROTAC model. Each surface represents the amount by which a conservation law is violated (purple, orange); the curve where each surface intersects the horizontal plane $Z=0$ (grey) corresponds to the set of system states where each conservation law is satisfied individually; the intersection point of the two curves (red dot) corresponds to the system solution, which satisfies both conservation laws simultaneously. Right: Validation of the exact steady state solution obtained from (14) (solid) vs. the numerical steady state solution of the differential Eq. (4) (dashed) (Color figure online)

The steady state solution for PROTACs is convoluted due to the complexity associated with three-body binding kinetics. In fact free target $\text {T}$ and free ligase $\text {L}$ are obtained simultaneously by numerically solving the following non-linear system of conservation laws for PROTAC and E3 ligase^1^:

14 $$\begin{aligned} {\left\{ \begin{array}{ll} \text {L}_{tot}(\text {T},\text {L}) = \text {L}+ \text {PL}(\text {T},\text {L}) + \text {TPL}(\text {T},\text {L}) = \text {L}_0\\ \text {P}_{tot}(\text {T},\text {L}) = \text {P}(\text {T},\text {L}) + \text {PT}(\text {T},\text {L}) + \text {PL}(\text {T},\text {L}) + \text {TPL}(\text {T},\text {L}) = \text {P}_0, \end{array}\right. } \end{aligned}$$

where $\text {P}$, $\text {PL}$, $\text {PT}$ and $\text {TPL}$ are all known calculated functions of $\text {T}$ and $\text {L}$ encapsulating surrogates of the original mechanistic parameters (Appendix D.1). Solving (14) means finding the one pair $\left( \text {T}^{\star }, \text {L}^{\star } \right)$ among all possible system states that does not violate the conservation laws, i.e., if we call $Z_1(\text {T},\text {L}) = \text {L}_{tot}(\text {T},\text {L}) - \text {L}_0$ and $Z_2(\text {T},\text {L}) = \text {P}_{tot}(\text {T},\text {L}) - \text {P}_0$ the amount by which each conservation law is violated, the solution to (14) will satisfy $Z_1( \text {T}^{\star }, \text {L}^{\star })=0$ and $Z_2( \text {T}^{\star }, \text {L}^{\star })=0$ simultaneously. Figure 4 provides a visual example: $Z_1$ and $Z_2$ are surfaces whose shape is dictated by the mechanistic parameters; the intersection between the two surfaces and the horizontal plane $Z=0$ corresponds to the pair $\left( \text {T}^{\star }, \text {L}^{\star } \right)$ that satisfies (14). Once $\text {T}^{\star }$ and $\text {L}^{\star }$ are calculated numerically, all the remaining states as well as the total remaining target

15 $$\begin{aligned} \text {T}_{tot}(\text {T},\text {L}) = \text {T}+ \text {PT}(\text {T},\text {L}) +\text {TPL}(\text {T},\text {L}), \end{aligned}$$

which is typically available through measurements, can be easily obtained through the exact expressions (D25a), (D25b), (D25c) (Appendix D.1). In particular, the total remaining target as a fraction of the baseline reads as:

16 $$\begin{aligned} \begin{aligned}&\widehat{\text {T}}_{tot}(\text {T},\text {L}) = \dfrac{k_{\text {deg}}}{k_{\text {PRO}}} + \left( 1- \dfrac{k_{\text {deg}}}{k_{\text {PRO}}}\right) \dfrac{k_{\text {deg}}}{k_{\text {syn}}} \text {T}+ \\&\left( 1 - \dfrac{k_{\text {MD}}}{k_{\text {PRO}}}\right) \dfrac{k_{\text {deg}}}{k_{\text {syn}}} \left\{ - \dfrac{k^{\text {T}}_{\text {on}}}{k^{\text {L}}_{\text {on}}}\dfrac{k^{\text {PT}}_{\text {off}}}{k^{\text {PT}}_{\text {on}}}\dfrac{k_{\text {deg}}}{k_{\text {PRO}}}\text {T}^2 \right. \\&\quad \left. + \dfrac{k^{\text {PT}}_{\text {off}}}{k^{\text {PT}}_{\text {on}}} \dfrac{k_{\text {syn}}}{k_{\text {PRO}}}\text {L}- \dfrac{k^{\text {PT}}_{\text {off}}}{k^{\text {PT}}_{\text {on}}} \dfrac{k_{\text {deg}}}{k_{\text {PRO}}}\text {L}\cdot \text {T}+\right. \\&\left[ \dfrac{k^{\text {T}}_{\text {on}}}{k^{\text {L}}_{\text {on}}}\dfrac{k^{\text {PT}}_{\text {off}}}{k^{\text {PT}}_{\text {on}}}\dfrac{k_{\text {syn}}}{k_{\text {PRO}}} - \left( \dfrac{k_{\text {deg}}}{k^{\text {PL}}_{\text {on}}}+ \dfrac{k^{\text {PL}}_{\text {off}}}{k^{\text {PL}}_{\text {on}}}\dfrac{k_{\text {deg}}}{k_{\text {PRO}}}\right) \dfrac{k^{\text {L}}_{\text {off}}}{k^{\text {L}}_{\text {on}}}\dfrac{k^{\text {T}}_{\text {on}}}{k^{\text {PT}}_{\text {on}}}\right. \\&\quad \left. -\dfrac{k^{\text {L}}_{\text {off}}}{k^{\text {L}}_{\text {on}}}\dfrac{k^{\text {T}}_{\text {on}}}{k^{\text {PL}}_{\text {on}}}\dfrac{k^{\text {PT}}_{\text {off}}}{k^{\text {PT}}_{\text {on}}}\dfrac{k_{\text {deg}}}{k_{\text {PRO}}}\right] \text {T}+\\&\left. \dfrac{k^{\text {L}}_{\text {off}}}{k^{\text {L}}_{\text {on}}}\left( \dfrac{k^{\text {PL}}_{\text {off}}}{k^{\text {PL}}_{\text {on}}}\dfrac{k_{\text {syn}}}{k_{\text {PRO}}} + \dfrac{k_{\text {syn}}}{k^{\text {PL}}_{\text {on}}}\right) \dfrac{k^{\text {T}}_{\text {on}}}{k^{\text {PT}}_{\text {on}}}\right. \\&\quad \left. + \dfrac{k^{\text {L}}_{\text {off}}}{k^{\text {L}}_{\text {on}}}\dfrac{k^{\text {T}}_{\text {on}}}{k^{\text {PL}}_{\text {on}}}\dfrac{k^{\text {PT}}_{\text {off}}}{k^{\text {PT}}_{\text {on}}}\dfrac{k_{\text {syn}}}{k_{\text {PRO}}} \right\} \bigg / \left\{ \text {L}^2 + \dfrac{k^{\text {T}}_{\text {on}}}{k^{\text {L}}_{\text {on}}}\text {L}\cdot \text {T}+ \right. \\&\left[ \dfrac{k^{\text {L}}_{\text {off}}}{k^{\text {L}}_{\text {on}}}\dfrac{k^{\text {T}}_{\text {on}}}{k^{\text {PL}}_{\text {on}}} + \left( \dfrac{k^{\text {T}}_{\text {off}}}{k^{\text {PT}}_{\text {on}}} + \dfrac{k^{\text {PT}}_{\text {off}}}{k^{\text {PT}}_{\text {on}}}\dfrac{k_{\text {MD}}}{k_{\text {PRO}}} + \dfrac{k_{\text {MD}}}{k^{\text {PT}}_{\text {on}}}\right) \right] \text {L}+ \\ {}&\left[ -\dfrac{k^{\text {T}}_{\text {off}}+k_{\text {MD}}}{k^{\text {L}}_{\text {on}}}\dfrac{k^{\text {T}}_{\text {on}}}{k^{\text {PT}}_{\text {on}}} + \dfrac{k^{\text {T}}_{\text {on}}}{k^{\text {L}}_{\text {on}}}\left( \dfrac{k^{\text {T}}_{\text {off}}}{k^{\text {PT}}_{\text {on}}} + \dfrac{k^{\text {PT}}_{\text {off}}}{k^{\text {PT}}_{\text {on}}}\dfrac{k_{\text {MD}}}{k_{\text {PRO}}} + \dfrac{k_{\text {MD}}}{k^{\text {PT}}_{\text {on}}}\right) \right] \text {T}\\&\left. - \dfrac{k^{\text {L}}_{\text {off}}}{k^{\text {L}}_{\text {on}}}\dfrac{k^{\text {T}}_{\text {on}}}{k^{\text {PT}}_{\text {on}}} \left( \dfrac{k^{\text {T}}_{\text {off}}}{k^{\text {PL}}_{\text {on}}} - \dfrac{k^{\text {PL}}_{\text {off}}}{k^{\text {PL}}_{\text {on}}}\dfrac{k_{\text {MD}}}{k_{\text {PRO}}} \right) \right. \\&\quad \left. + \dfrac{k^{\text {L}}_{\text {off}}}{k^{\text {L}}_{\text {on}}}\dfrac{k^{\text {T}}_{\text {on}}}{k^{\text {PL}}_{\text {on}}}\left( \dfrac{k^{\text {T}}_{\text {off}}}{k^{\text {PT}}_{\text {on}}} + \dfrac{k^{\text {PT}}_{\text {off}}}{k^{\text {PT}}_{\text {on}}}\dfrac{k_{\text {MD}}}{k_{\text {PRO}}} + \dfrac{k_{\text {MD}}}{k^{\text {PT}}_{\text {on}}}\right) \right\} \end{aligned} \end{aligned}$$

and it can be verified that it is equivalent to the numerical steady state solution of the differential Eq. (4) (Fig. 4).

#### Global sensitivity analysis

**Fig. 5:**
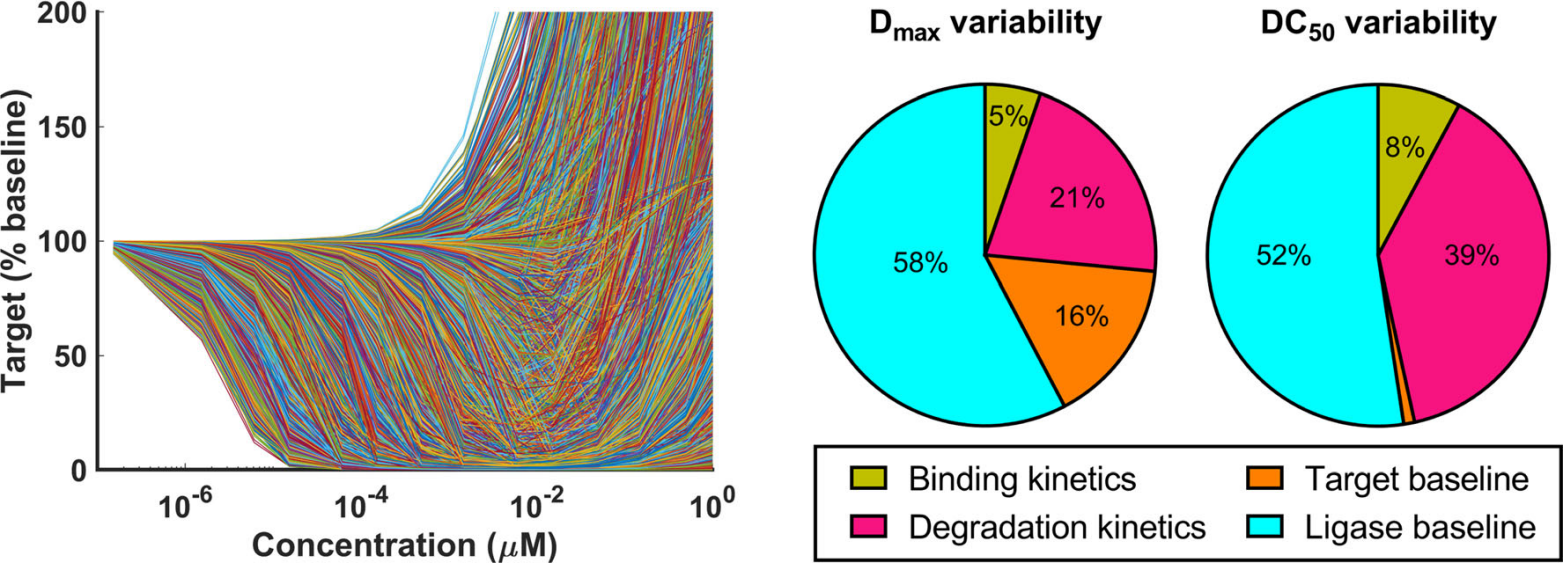
Global sensitivity analysis of the PROTAC mechanistic model. Simulated dose-responses associated with sampled parameter space (left) and variance decomposition of $\text {DC}_{50}$ and $\text {D}_{max}$ via Sobol indices (right)

Global sensitivity analysis was performed on the mechanistic model (4) assuming uniform distribution of the parameters over the ranges listed in Table 2. In order to reduce the exponentially high computational cost of GSA on a high-dimensional parameter space, no cooperativity was assumed ($\alpha = 1$, or $k^{\text {PL}}_{\text {on}}= k^{\text {T}}_{\text {on}}$, $k^{\text {PT}}_{\text {on}}= k^{\text {L}}_{\text {on}}$, $k^{\text {PL}}_{\text {off}}= k^{\text {T}}_{\text {off}}$, $k^{\text {PT}}_{\text {off}}= k^{\text {L}}_{\text {off}}$). Smolyak sparse quadratures of level 6 were used, corresponding to 242815 sampled parameter combinations. Sobol indices of $\text {DC}_{50}$ and $\text {D}_{max}$ were calculated for each parameter from the corresponding simulated dose-responses and grouped by category to ease the interpretation (Fig. 5). More precisely, the fractions of variability due to binding kinetics, degradation kinetics, and target baseline sum up the contribution of the Sobol indices of on/off rates ($k^{\text {T}}_{\text {on}}$, $k^{\text {T}}_{\text {off}}$, $k^{\text {L}}_{\text {on}}$, $k^{\text {L}}_{\text {off}}$), degradation rates ($k_{\text {MD}}$, $k_{\text {PRO}}$), and endogenous target synthesis/turnover ($k_{\text {syn}}$, $k_{\text {deg}}$), respectively. Most of the variability of both $\text {DC}_{50}$ and $\text {D}_{max}$ is due to changes in ligase baseline ($>50\%$), followed by degradation efficiency ($\sim 20-40\%$) and target baseline ($\sim 20\%$). Based on the selected parameter range and distribution, binding kinetics contributes only minimally to the overall response variability ($\sim 5\%$).

**Table 2:** Parameter ranges for global sensitivity analysis of PROTAC mechanistic model

Description	Parameter	Range
Target or Ligase baselines	$\text {T}_0$, $\text {L}_0$	$\left[ 0.1 nM, 1 \mu M \right]$
Target or Ligase ON rate	$k^{\text {T}}_{\text {on}}$, $k^{\text {L}}_{\text {on}}$, $k^{\text {PT}}_{\text {on}}$, $k^{\text {PL}}_{\text {on}}$	$\left[ 10^5, 10^7 \right] \ M \cdot s$
Target or Ligase OFF rate	$k^{\text {T}}_{\text {off}}$, $k^{\text {L}}_{\text {off}}$, $k^{\text {PT}}_{\text {off}}$, $k^{\text {PL}}_{\text {off}}$	$\left[ 10^{-3}, 10^{-1} \right] /s$
Ternary complex half-life	$\ln (2)/k_{\text {PRO}}$	$\left[ 0.1, 1000 \right] s$
Cooperativity	$\alpha$	1 (fixed)

## Discussion

### Monovalent degraders

#### Compound optimization

**Fig. 6:**
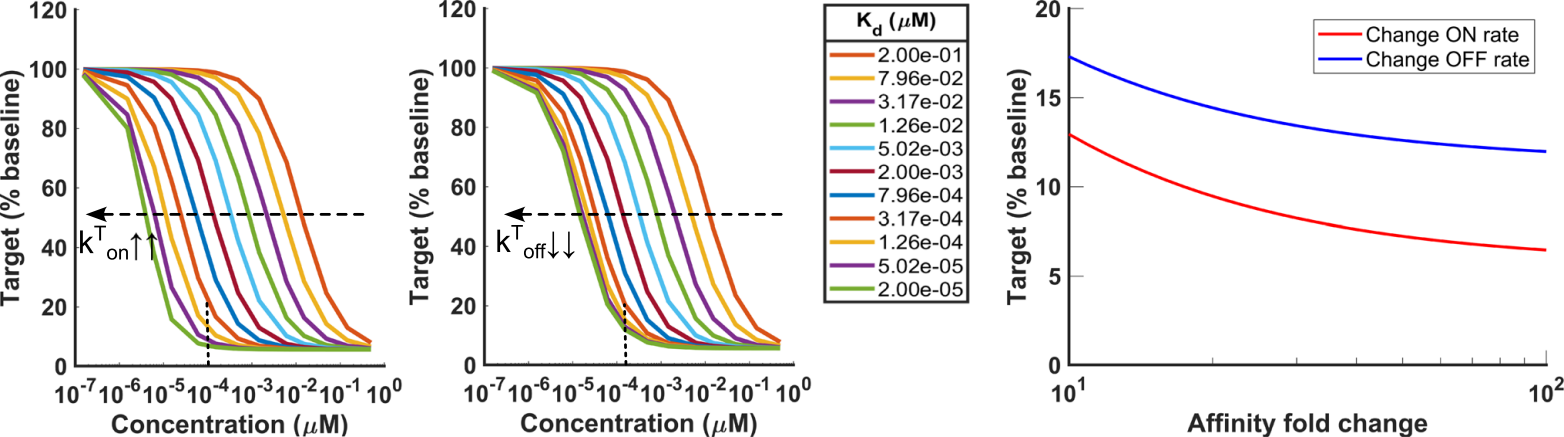
Local sensitivity analysis of degradation by MDs to changes in binding equilibrium constant ($K_d$) driven by either on-rate (left) or off-rate (center). For the same change in affinity at a fixed concentration (dashed vertical line) the asymptotic response is different depending on whether the change in affinity is due to a variation in on- (red) vs off-rate (blue) (right) (Color figure online)

Binding equilibrium constants ($K_d=k^{\text {T}}_{\text {off}}/k^{\text {T}}_{\text {on}}$) are often used to drive SAR of small molecules. While they have proved useful in practice [47–50], from a mechanistic viewpoint they can only provide limited understanding of the major drivers of pharmacology:

1. **On/off rates matter** *individually*. Consider the case where $k^{\text {T}}_{\text {off}}$ is minimized vs. the case where $k^{\text {T}}_{\text {on}}$ is maximized. In both cases the overall effect on thermodynamics is that the binding affinity increases (i.e. $K_d$ decreases), however the same variation in affinity can actually result in different degradation profiles. This is particularly evident in the two extreme scenarios where $k^{\text {T}}_{\text {off}}$ is extremely low (namely $k^{\text {T}}_{\text {off}}\rightarrow 0^+$, as it could be the case, e.g., of a covalent binder) or when $k^{\text {T}}_{\text {on}}$ is much greater than $k^{\text {T}}_{\text {off}}+k_{\text {MD}}$ (namely $k^{\text {T}}_{\text {on}}\rightarrow +\infty$). The definition in (13) clearly shows that by reducing $k^{\text {T}}_{\text {off}}$ one can only lower $\text {DC}_{50}$ to $(1-\widehat{D}_{max})k_{\text {MD}}/k^{\text {T}}_{\text {on}}$, whereas by increasing $k^{\text {T}}_{\text {on}}$ there is virtually no limit to how potent a compound can be made (namely, up to $\text {DC}_{50}=0$). Analogously for $\widehat{D}_{max}$, from Eq. (11) we can clearly see that, for the same degradation efficiency ($k_{\text {MD}}$), ever faster on-rates will reduce the target to a minimum relative amount equivalent to the ratio of endogenous and MD degradation rate (Eq. (17a)); differently, ever slower off-rates will spare a portion of residual protein that is inversely proportional to the amount of free drug at steady state (Eq. (17b)):
  17a $$\begin{aligned} \dfrac{\text {T}_{tot}}{\text {T}_0}(\text {D})&\xrightarrow {k^{\text {T}}_{\text {on}}\rightarrow +\infty } \text {T}^{\text {ON}} = \dfrac{k_{\text {deg}}}{k_{\text {MD}}} \end{aligned}$$
  17b $$\begin{aligned} \dfrac{\text {T}_{tot}}{\text {T}_0}(\text {D})&\xrightarrow {k^{\text {T}}_{\text {off}}\rightarrow 0^+} \text {T}^{\text {OFF}} = \dfrac{k_{\text {deg}}}{k_{\text {MD}}} + \left( 1 - \dfrac{k_{\text {deg}}}{k_{\text {MD}}} \right) \cdot \dfrac{\dfrac{k_{\text {deg}}}{k^{\text {T}}_{\text {on}}} }{\text {D}+ \dfrac{k_{\text {deg}}}{k^{\text {T}}_{\text {on}}}}. \end{aligned}$$
  In formulae, notice that the limit $\text {T}^{\text {OFF}}$ in (17b) is equal to $\text {T}^{\text {ON}}$ (17a) added by a residual term, which clearly shows that optimizing off rates leads to inferior degradation (Fig. 6).
2. **Maximizing on-rates is the most efficient way to maximize binary complex degradation via binding kinetics**. Minimizing (11) solely via binding kinetics entails minimizing the residual term. Because of its functional structure, this can only be achieved by making the ratio $\dfrac{k^{\text {T}}_{\text {off}}+k_{\text {MD}}}{k^{\text {T}}_{\text {on}}}$ as close to 0 as possible, i.e., by maximizing $k^{\text {T}}_{\text {on}}$.
3. **Binding and degradation optimization is coupled**. Eq. (11) highlights how the residual term that prevents a MD from achieving maximal degradation ($1-k_{\text {deg}}/k_{\text {MD}}$) is governed not only by the binding equilibrium constant $k^{\text {T}}_{\text {off}}/k^{\text {T}}_{\text {on}}$ but also by the ratio of binary complex *degradation* rate to the *binding* on-rate ($k_{\text {MD}}/k^{\text {T}}_{\text {on}}$), which parameters are clearly different in nature (pharmacology vs. chemistry). This shows that binding and degradation cannot be thought of separately. In other words, relatively slow binding can be efficient enough for a slow degrader, but on the other hand a fast degrader will require fast binding to deliver its full potential. For many years medicinal chemist have focused on increasing the drug-target residence time by means of optimizing off-rates [47, 48]. While that approach has been successful, this analysis suggests that for degraders optimizing on-rates is a more efficient approach. Copeland et al already highlighted that optimization of the target residence time (i.e. off-rate) may have limited utility in cases where the rate of new protein synthesis (and degradation) plays an important role [47, 48]. Multiple authors [47, 48, 51] mention an upper limit to on-rates ($10^8-10^9 M^{-1}S^{-1}$) given by the rate of diffusion of the two binding partners in physiological solutions, however Schoop et al [51] show that several discovery programs have not yet achieved such upper limit, indicating that there may still be room to further increase on-rates. Medicinal chemists have been confronted with the question of whether it is possible to design on-rates for a given target within a given compound series but Schoop has shown different compound series of distinct targets which display significant differences in their on-rates. Obviously the difficulty facing medicinal chemist is establishing general $k_{on}$ optimization strategies. Optimization of on-rates through SAR has been limited and mostly empirical due to the difficulty to predict the physico-chemical steps involved in receptor-ligand association (protein conformational re-arrangements, protein desolvation and molecular orbital orientation) [47, 48, 51].
4. **Improving degradation efficiency is necessary to increase maximal degradation, and sufficient but not necessary to improve degradation potency** Because $\text {D}_{max}$ only depends on the (endogenous and) MD degradation rate via (13), the only way to increase maximal degradation is by increasing $k_{\text {MD}}$. Moreover, because degradation potency directly depends on $\text {D}_{max}$ (13), improving degradation efficiency ($k_{\text {MD}}$) always results in higher potency (lower $\text {DC}_{50}$) as well. However, the opposite is not necessarily true: an improvement in potency driven by optimized binding kinetics (faster $k^{\text {T}}_{\text {on}}$ or slower $k^{\text {T}}_{\text {off}}$) has no impact whatsoever on $\text {D}_{max}$.
5. **Complete degradation can only be reached asymptotically; faster turnover proteins are harder targets.** It is impossible to achieve net $100\%$ degradation because $\widehat{\text {T}}_{min}= k_{\text {deg}}/k_{\text {MD}}$ is always strictly positive, no matter how small the ratio. Moreover, faster protein turnover requires higher degradation efficiency to maintain a given level of target knock-down.

In conclusion, optimizing binding affinities is a simplistic approach that can lead to ambiguous outcomes because the response is sensitive to on and off rates individually, and binding kinetics should be optimized relatively to degradation kinetics (and conversely).

#### Model identifiability and data requirements

Although analyzing the *fraction* of total remaining target relative to baseline would be more relevant because truly representative of the available experimental data, for the sake of simplicity we now focus on the expression of the free target *concentration*. In Appendix B.3 we show that the same conclusions hold true for the fraction of total remaining target.

If we lump the coefficients in (10) into a new synthetic parametrization to highlight the equation structure (Eqs. (18)-(19)) it becomes evident that while the original mechanistic model (1) is governed by *five* parameters ($k_{\text {syn}}$, $k_{\text {deg}}$, $k^{\text {T}}_{\text {on}}$, $k^{\text {T}}_{\text {off}}$, $k_{\text {MD}}$), the corresponding steady state (10) only features *three* independent degrees of freedom ($\alpha$, $\beta$, $\gamma$, see Appendix B.3), which are *surrogates* of the original mechanistic parameters:

18 $$\begin{aligned} - \alpha \cdot \text {T}+ \alpha \cdot \gamma \cdot \dfrac{1}{\text {T}} + \alpha - \beta \cdot \gamma = \text {D}_0, \end{aligned}$$

where

19 $$\begin{aligned} \begin{aligned} \alpha&= \dfrac{k_{\text {syn}}}{k_{\text {MD}}} \\ \beta&= \dfrac{k_{\text {deg}}}{k_{\text {MD}}} \\ \gamma&= \dfrac{k^{\text {T}}_{\text {off}}+k_{\text {MD}}}{k^{\text {T}}_{\text {on}}}. \end{aligned} \end{aligned}$$

This apparently simple observation is extremely informative on model identifiability and data requirements:

1. If the only available data is free target concentration at steady state $\text {T}$, the surrogate parameters $\alpha$, $\beta$ and $\gamma$ can be uniquely estimated, but *none* of the original mechanistic parameters can be uniquely identified.
2. If, in addition to point (1), the endogenous degradation rate $k_{\text {deg}}$ is available, the MD degradation rate $k_{\text {MD}}$ can be obtained from (19) as
  20 $$\begin{aligned} k_{\text {MD}}= \dfrac{k_{\text {deg}}}{\beta } \end{aligned}$$
  and consequently the endogenous synthesis rate $k_{\text {syn}}$ and protein baseline $\text {T}_0$ as
  21 $$\begin{aligned} k_{\text {syn}}= k_{\text {MD}}\cdot \alpha , \qquad \text {T}_0= \dfrac{k_{\text {syn}}}{k_{\text {deg}}}. \end{aligned}$$
3. If, in addition to points (1) and (2), the binding equilibrium constant $K_d= k^{\text {T}}_{\text {off}}/k^{\text {T}}_{\text {on}}$ is available, also the individual on/off rates can be calculated as
  22 $$\begin{aligned} k^{\text {T}}_{\text {on}}= \dfrac{k_{\text {MD}}}{\gamma - K_d}, \qquad k^{\text {T}}_{\text {off}}= K_d\cdot k^{\text {T}}_{\text {on}}. \end{aligned}$$

#### Example: baseline estimation from degradation data

**Fig. 7:**
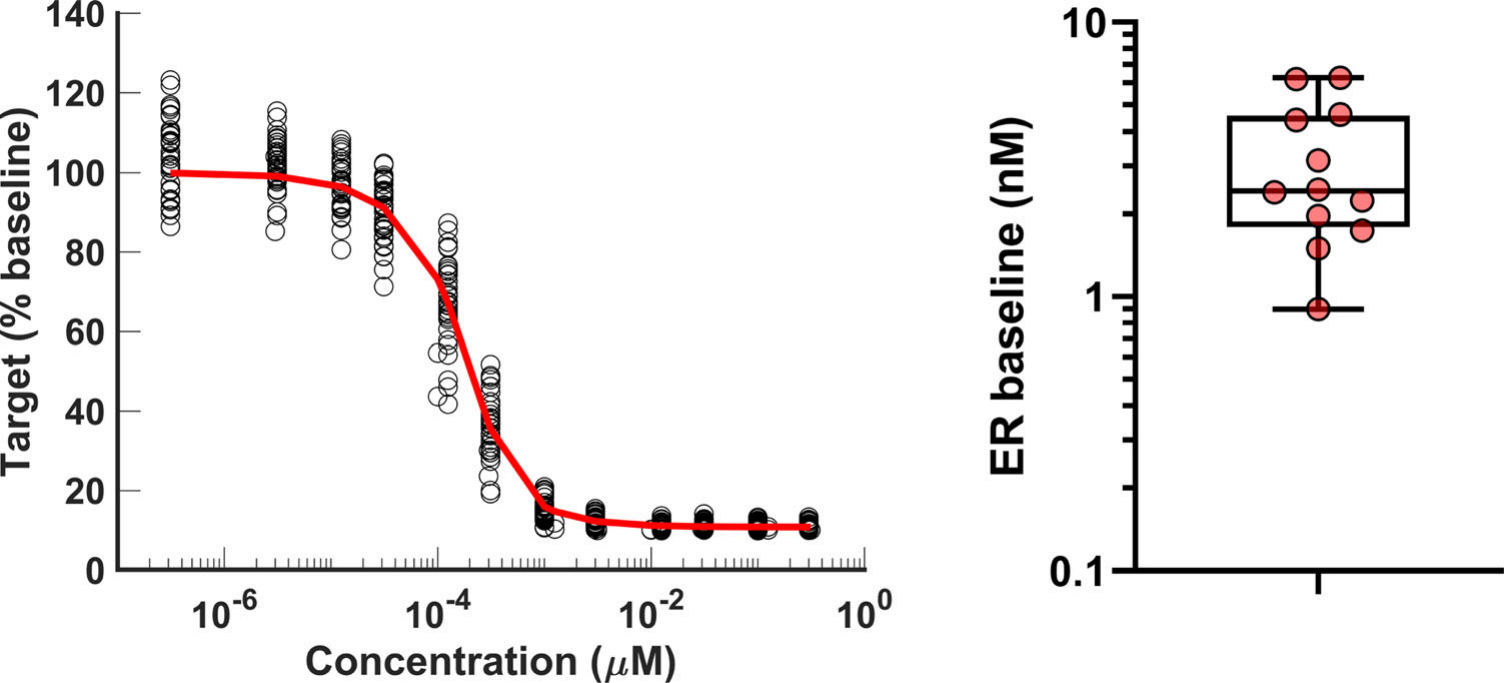
Example of MD dose-response with mechanistic model fit (left) and estimated baselines (right). Each point represents the baseline estimate obtained by fitting the dose-response of 12 different SERDs individually

Eq. (B14) in Appendix B.3 describing the fraction of *total* remaining target at steady state was fitted in Matlab to the *in vitro* degradation dose-response of 12 SERDs to obtain the surrogate parameters $\alpha$, $\beta$, $\gamma$ (An example is shown in Fig. 7, left). The measured endogenous fractional turnover rate of ER$\alpha$ in MCF-7 ($k_{\text {deg}}=0.15$/h, Appendix C) was used to calculate the MD degradation rate $k_{\text {MD}}$ via (20).

Then, ER baseline was estimated from each compound from (21) with an interquartile range of $1.86-4.51$nM (Fig. 7, right), which is within published concentrations of ER for MCF-7 cells [52, 53].

### PROTACs

#### Compound optimization

We have shown that optimizing MDs based on binding affinity alone is sub-optimal because the same change can produce different effects on degradation, depending on whether such variation is due to slower/faster on-rate vs off-rate. Differences in the effect can be traced back to binding kinetics and degradation kinetics being coupled or, more precisely, to the *proportion* of MD degradation rate $k_{\text {MD}}$ and on-rate $k^{\text {T}}_{\text {on}}$. In this respect, although drawing similar conclusions for PROTACs is less straightforward due to the complexity of the steady state solution, understanding parameter dependencies (i.e., *proportions* of mechanistic parameters or which ones matter *with respect to* which others) can provide at least general guidelines on the most critical aspects of optimization.

**Fig. 8:**
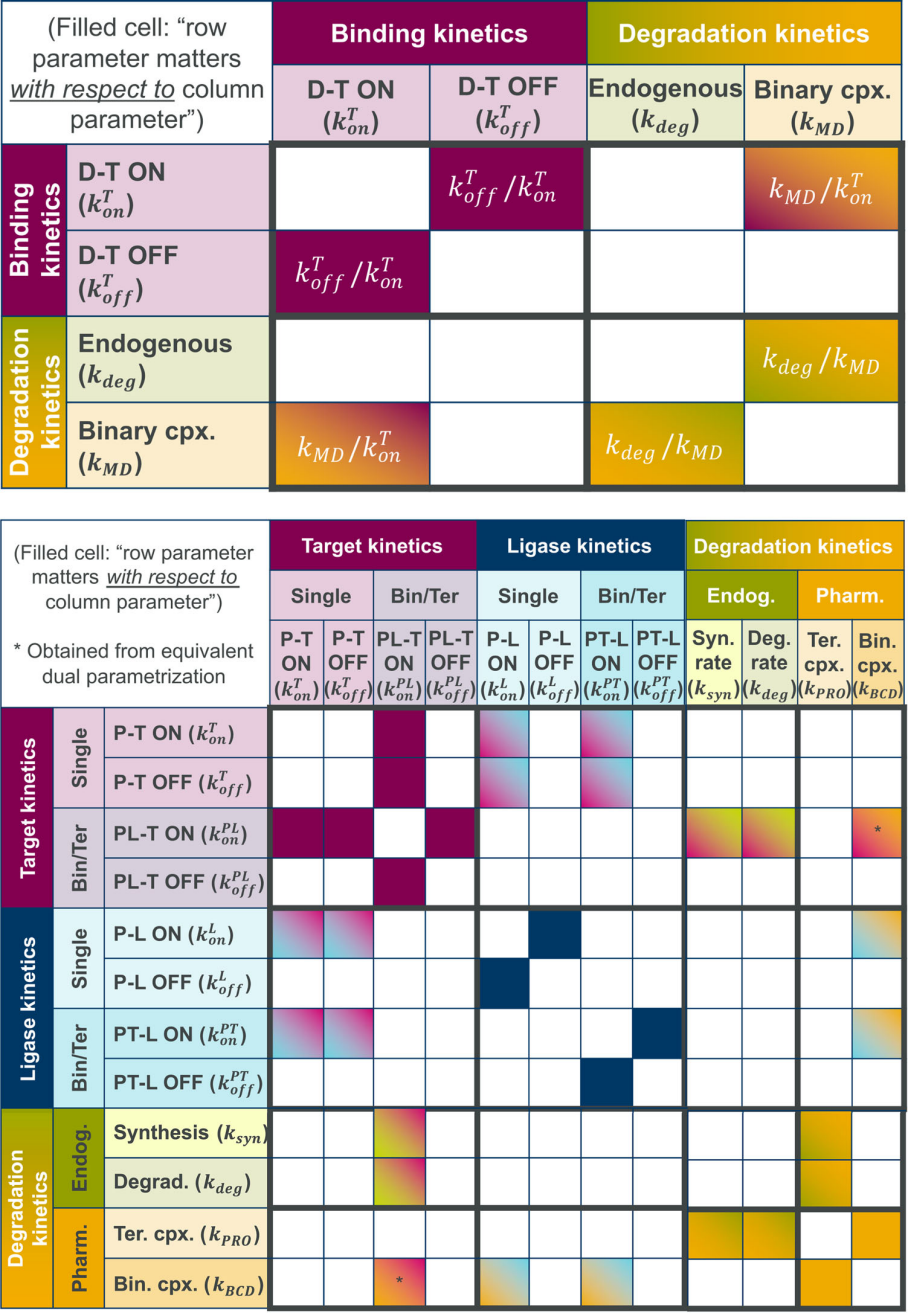
Tables of parameters interactions emerging from the exact steady state solution of MD (top) and PROTAC (bottom) mechanistic models. A filled cell in row *r*, column *c* indicates that the parameters in row *r* and column *c* matter *relatively* to each other. Relationships marked with an asterisk refer to an equivalent dual parametrization of the exact solution (not shown, see Appendix D) and are included for completeness

The core of PROTACs inter-parameter relationships and proportions is encoded in coefficients $c_{ij}$’s and $t_{ij}$’s (Appendix D.2). Rather than analyzing the expression of each individual coefficient, we will summarize such relationships in a table where both rows and columns correspond to kinetics, pharmacological or biological parameters, and the cell in a given row *r*, column *c* is filled if the *proportion* between the two parameters in the corresponding row and column has an effect on steady state degradation (i.e., if they appear in a ratio in the coefficients). For instance, the MD solution (10) contains the ratios $k^{\text {T}}_{\text {off}}/k^{\text {T}}_{\text {on}}$, $k_{\text {deg}}/k_{\text {MD}}$, $k_{\text {MD}}/k^{\text {T}}_{\text {on}}$, which correspond to the table pattern in Fig. 8 (top). The non-empty fingerprint of the top-right or bottom-left off-diagonal blocks clearly marks the coupling between binding and degradation kinetics previously noted ($k_{\text {MD}}/k^{\text {T}}_{\text {on}}$). Furthermore, while the top-left diagonal block encodes the binding affinity $k^{\text {T}}_{\text {off}}/k^{\text {T}}_{\text {on}}$, the bottom-right one captures the maximum achievable degradation (17a), given by the proportion between the endogenous and MD degradation rates $k_{\text {deg}}/k_{\text {MD}}$, thus coupling biology with pharmacology.

The fingerprint of the PROTACs solution is shown in Fig. 8 (bottom). As for MDs, the table is partitioned into binding and degradation kinetics, however the former block is further sub-divided into target and E3 ligase kinetics of single species or binary complexes, for a total of $2 \times 2$ sub-blocks of size $4 \times 4$.

1. **Binding and degradation optimization is coupled**. First and foremost, as in the previous example it is evident that the matrix pattern is *not* block-diagonal, i.e., binding and degradation kinetics are intertwined.
2. **PROTACs are more than just the sum of their components.** More specifically, even the top-left two-by-two target/ligase binding kinetics block is not block-diagonal: this means that PROTACs-induced response is the result of not only target and ligase kinetics *individually*, but also of the *interplay* between the two. For instance, target-PROTAC on-rate ($k^{\text {T}}_{\text {on}}$) matters *relatively* to ligase-PROTAC on rate ($k^{\text {L}}_{\text {on}}$). This fact underscores that PROTACs design goes way beyond the optimization of its individual components (E3 ligase and target warheads), i.e., optimizing each portion independently may not guarantee an optimal compound overall.
3. **On/off rates matter** *individually*. Each on/off rate plays a unique and specific role in relationship with the rest of the parameters (the fingerprint of each row or column is specific to each parameter). As for MDs, even more so for PROTACs this implies that changes not only to each binding affinity, but also on cooperativity can produce a different effect depending on whether on- vs off-rates are changed. Therefore studying how the response varies as cooperativity changes is an ill-posed question as it is for binding affinities.
4. **Increased cooperativity via faster on-rates promotes degradation.** As described above, an excess of $\text {PT}$ (or $\text {PL}$) binary complex can impair efficient degradation up to causing protein stabilization. The exact solution fingerprint of $k_{\text {MD}}$ shows that $\text {PT}$ accumulation can be mitigated via binding kinetics in two ways: by favoring the ligase pathway via faster PROTAC-ligase on rates ($k_{\text {MD}}/k^{\text {L}}_{\text {on}}$ ratio), or by increasing cooperativity via on rates ($k_{\text {MD}}/k^{\text {PL}}_{\text {on}}$, $k_{\text {MD}}/k^{\text {PT}}_{\text {on}}$ ratios).

#### Model identifiability and structural data requirements

**Model observability**

Despite the complexity of calculations and resulting expressions, the compact notation in Eq. (14) is key to highlight a structural feature of the system with implications on minimal data requirements. First of all, the E3 ligase baseline $\text {L}_0$ is an input to the system, just as much as the drug concentration $\text {P}_0$, therefore experimental knowledge of it is a prerequisite. Secondly, if we first consider the simpler case of MDs, expression (18) clearly shows that observations of only *one* species (the remaining free target $\text {T}$ following a corresponding given dose $\text {D}_0$) is necessary and sufficient to infer the remaining states of the system (binary complex $\text {TD}$ and free MD $\text {D}$ from (B4a) and (B5) in Appendix B, respectively) and possibly estimate the surrogate parameters (provided enough distinct observations). Differently, for PROTACs, Eqs. (14) feature *two* independent variables ($\text {T}$ and $\text {L}$) for each given concentration $\text {P}_0$ which cannot be further reduced. This suggests that observations of at least *two* different states are required in order to infer the state of the whole system and possibly estimate its surrogate parameters. Such two species do not necessarily need to be free target and free ligase: any other pair of observations (except for total ligase and total PROTAC, which are conserved) is viable because $\text {T}$ and $\text {L}$ can be retrieved from relationships (D23a), (D25a)-(D25c) (Appendix D.1) to then solve (14). For instance, if the concentrations $\text {T}^\star$ and $\text {T}_{tot}^\star$ were observed for free target $\text {T}$ and total target $\text {T}_{tot}$, free ligase concentration $\text {L}$ could be calculated by solving $\text {T}^\star + \text {PT}(\text {L};\text {T}^\star ) +\text {TPL}(\text {L};\text {T}^\star )=\text {T}_{tot}^\star$, where the notation $(\text {L}; \text {T}^\star )$ indicates functional dependence on ligase, given the target concentration, and the exact functional relationships of $\text {PT}$ and $\text {TPL}$ are known from (D23a) and (D25c). Analogously, if total target $\text {T}_{tot}^\star$ and ternary complex $\text {TPL}^\star$ were observed, target and ligase concentrations could be retrieved by solving Eq. (15) and (D23a) for $\text {T}$ and $\text {L}$ simultaneously, given $\text {T}_{tot}^\star$ and $\text {TPL}^\star$.

In summary, the exact solution structure practically implies that measuring total remaining target concentration *only* is insufficient to infer the state of the whole steady state system, which also requires knowledge of the E3 ligase baseline.

**Model identifiability**

While these conclusions characterize the steady state system observability, i.e., the ability of inferring the state of the whole system given a set of observations, understanding which surrogate parameters can be uniquely identified (such as $\alpha$, $\beta$ and $\gamma$ for MDs) is not as immediate. Ultimately, the steady state solution is characterized by 14 surrogate coefficients ($c_{ij}$’s and $t_{ij}$’s, Appendix D.2) which, alone, are insufficient to retrieve the original 11 mechanistic parameters. In fact, by constructing a Gröbner basis [54] it can be shown that only 9 of them are actually independent. This means that at least 2 mechanistic parameters (or corresponding surrogates) have to be measured to be able to retrieve all the others from 9 model coefficient estimates. For instance, if the endogenous degradation rate $k_{\text {deg}}$ (or the endogenous synthesis rate $k_{\text {syn}}$, or the binary complex degradation rate $k_{\text {MD}}$) *and* the PROTAC-target binding affinity $k^{\text {T}}_{\text {on}}/k^{\text {T}}_{\text {off}}$ are available from measurements, all the original mechanistic parameters can be calculated (Appendix D.3). The generalization of this conclusion to all possible sets of measurements from which it is possible to retrieve the mechanistic parametrization will be addressed in future work.

#### Data prioritization: impact of protein endogenous baselines on pharmacology

**Fig. 9:**
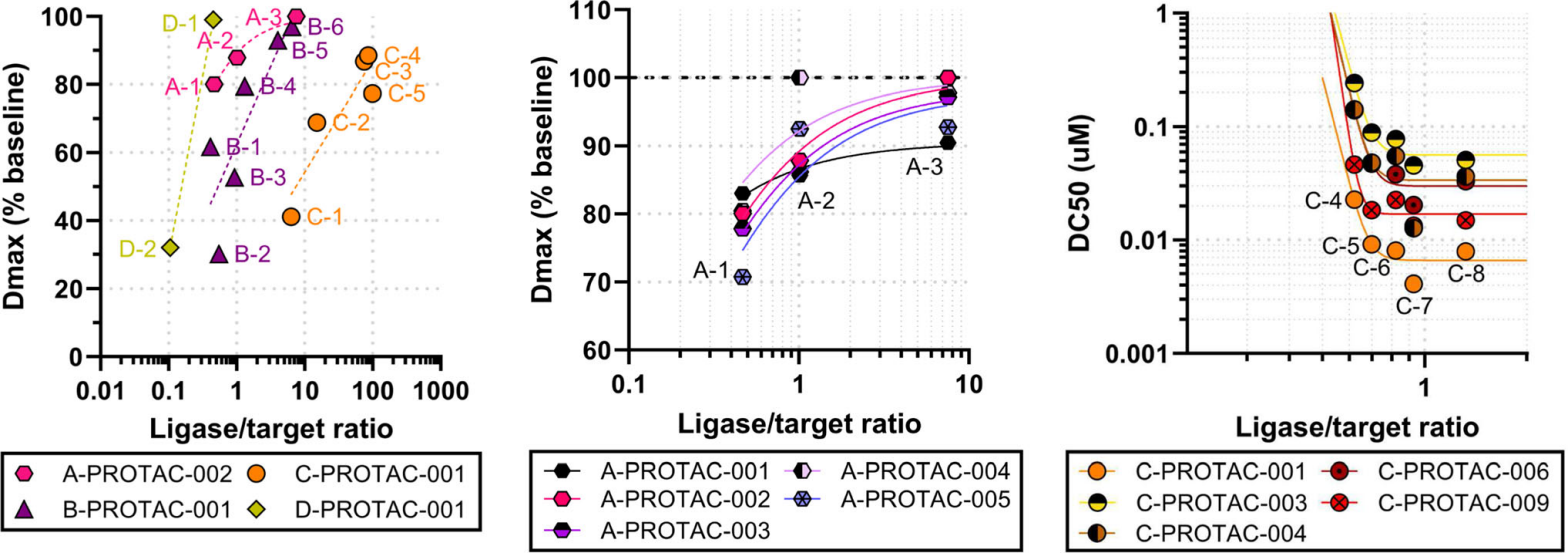
Correlation between $\text {D}_{max}$ and ratio of ligase and target baselines across different targets (left; only one compound per program is shown for easiness). $\text {D}_{max}$/baseline ratio correlation of several compounds targeting protein A (center). $\text {DC}_{50}$/baseline ratio correlation of several compounds targeting protein C (left)

While drawing firm guidelines on PROTACs optimization is challenging due to the complexity of the system, the outcome of global sensitivity analysis can be extremely valuable to guide data prioritization and model simplification. First and foremost, it is striking how most of the total response variability (up to $~75\%$) is not driven by chemical or pharmacological factors such as binding and degradation rates, which can indeed be optimized, rather by *biological* factors, i.e. ligase and target baselines, over which we have no control. This shows how critical it is to characterize the distribution of such parameters in the patient population and, consequently, to judiciously select the most appropriate pre-clinical patient representative tumour models [55]. More precisely, data collected from four different AstraZeneca PROTACs projects clearly show how $\text {D}_{max}$ correlates with the *ratio* of ligase and target baselines, i.e., the higher the ligase levels *relatively* to the target the higher the degradation (Fig. 9). It is to be noted that here baseline quantification is obtained from Western Blots and hence is only semi-quantitative. While efforts are in place to generate absolute quantification of protein levels in relevant models to confirm such relationship, the fact that a similar trend is observed across multiple targets and compounds from different chemical series is early evidence that such correlation may hold true in practice, both *in vitro* and *in vivo* [15]. Note that even within the same target space not all molecules may display the same type of correlation: a range of different slopes or $\text {D}_{max}$ may be observed. For instance, Fig. 9 (center) shows how A-PROTAC-001 displays a flatter profile with a lower $\text {D}_{max}$ compared to other compounds targeting the same protein of interest and ligase. What makes a PROTAC more or less sensitive to changes in baseline ratio is still unclear, and which profile is more desirable depends on the PKPD/E and toxicity relationship of the target. In this example, if $80-90\%$ maximal degradation is sufficient to drive efficacy then A-PROTAC-001 is preferable because it is expected to be efficacious in most of the relevant cell lines and hence ultimately in a wider set of baseline ratios (which may be observed in the target population). On the other hand, if nearly complete degradation is required for efficacy then A-PROTAC-002-005 may be preferable because they can achieve higher $\text {D}_{max}$ up to $\sim 100\%$. However, in this scenario characterizing the ligase and target baseline *distribution* in the patient population is even more critical: if the ratio is relatively low in *most* patients these PROTACs may be unable to achieve complete degradation due to the steep correlation with the baseline ratio, and as a result efficacy may be compromised. At the same time, though, a sharp slope may enable a better therapeutic index: if the relevant tox tissues display lower ratios compared to the patient representative tumour models a steeper relationship would entail degrading the target in the tumor while sparing it in normal tissues [15].

#### A pragmatic approach to modelling

This analysis implies an empirical approach to build a mathematical model of medium complexity with the potential of being truly predictive. While, on the one hand, the fully mechanistic model is over-parametrized and hence unlikely to be predictive, on the other hand traditional turnover models such as (9) are also unlikely to be predictive across patient representative tumour models because unable to capture differences in $\text {D}_{max}$ driven by changes in baselines. However, GSA of the former suggests that it is possible to “enrich” or “augment” the latter with data-driven components. More precisely, $\text {D}_{max}$ can be implemented as a (sigmoidal) function of the baseline ratio as inferred from the data rather than as a constant parameter, and the same can be done for $\text {DC}_{50}$ (Fig. 9, right). For instance, if $\rho$ is the ligase/target baseline ratio these two parameters can be described as

23 $$\begin{aligned} \text {D}_{max}(\rho )= & {} \dfrac{\rho ^\delta }{\rho ^\delta + \rho _{50}^\delta }, \qquad \nonumber \\ \text {DC}_{50}(\rho )= & {} (\text {DC}_{50, max}- \text {DC}_{50, min})e^{-\kappa \cdot \rho } + \text {DC}_{50, min} \end{aligned}$$

and used in Eq. (9). Clearly, this type of modelling requires generating dose-responses in multiple patient representative tumour models spanning a range of baseline ratios, which is more demanding than a simple dose-response but still less prohibitive and more easily accessible than individual on/off rates. Not only, it has the potential of predicting the response of new patient representative tumour models or tissues (or even patients) as they become of interest or available.

**Fig. 10:**
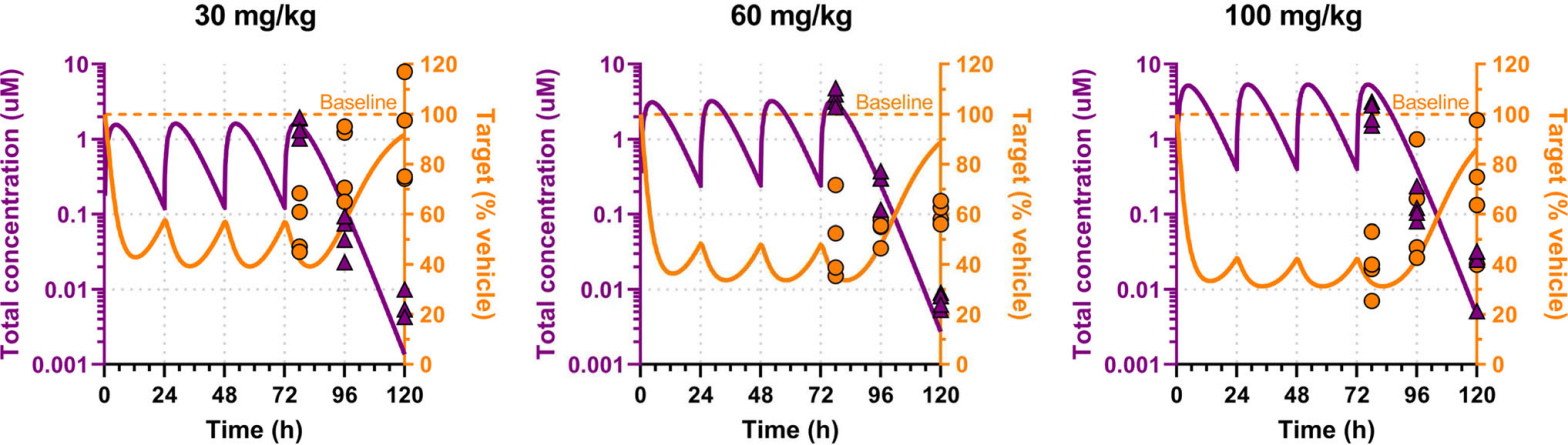
PK fit and PD predictions of *in vivo* degradation kinetics from *in vitro* indirect response model in the same cell line as the *in vivo* model by C-PROTAC-009 at different dose levels

Whenever baseline and/or dose-response data is unavailable or insufficient to fully parametrize relationships (23), it is critical to use the same *in vitro* cell line as the *in vivo* model to increase the chances of operating in biological settings with comparable baseline ratio $\rho$ (even without knowing its value) while mitigating the risk of a disconnect in $\text {D}_{max}$ and potency due to potential differences in baseline ratio. For instance, Fig. 10 shows how a PD model built on degradation time course data in model C-9 *in vitro*, driven by the plasma PK profile fitted *in vivo* in the same C-9 model, can successfully predict degradation kinetics *in vivo* at multiple dose levels.

## Conclusion

In this work we have shown the value of an integrated modelling approach for degraders which combines the benefits of traditional turnover models and fully mechanistic models to address two key project questions in drug discovery programs, i.e. (i) how to drive compound optimization and (ii) how to predict pharmacology across patient representative tumour models or the kinetics of *in vivo* degradation from *in vitro* data, ultimately to support dose selection and predictions.

Whenever an exact mechanistic steady state solution is available it can be utilized to precisely understand the role of each parameter on the response and hence which kinetic “knobs” (e.g. on/off rates) need to be tuned to achieve a desired pharmacological profile ($\text {D}_{max}$, $\text {DC}_{50}$). If such properties can be measured experimentally in high-throughput format they can directly inform and guide the design of novel compounds, otherwise it may be possible to obtain them indirectly from degradation data.

Following this approach we have shown how on/off and degradation rates are related to potency and maximal effect of monovalent degraders, and how such relationship can be used to suggest a compound optimization strategy based on faster association rather than slower dissociation. Moreover, the same relationship indicates which additional experimental data (e.g. endogenous protein turnover and binding affinity) should be collected to retrieve all the original mechanistic parameters, which we demonstrated for SERDs.

Whenever an exact steady state solution is not available, or when its complexity is prohibitive (such as for PROTACs), an empirical data-driven, mechanism-agnostic approach offers a sub-optimal yet more pragmatic option. Nevertheless, even complex exact steady state solutions can provide insight on the *type* of observations required to ensure the predictive capacity of a mechanistic approach. Specifically for PROTACs, the *structure* of the exact steady state solution suggests that the *total* remaining target at steady state, which is easily accessible experimentally, is insufficient to reconstruct the state of the whole system at equilibrium and observations on different states (such as binary/ternary complexes) are necessary. Not only, it highlights how the E3 ligase baseline levels are a direct *input* to the system – just as the compound concentration – and therefore should be measured beforehand in biological models of interest.

While parameter identifiability is challenging for PROTACs, global sensitivity analysis suggests that *both* target and ligase baselines (actually, their ratio) are the major sources of variability in the response of non-cooperative systems, which speaks to the importance of not only generating such data as early as possible in drug discovery, but also characterizing their *distribution* in the target patient population to maximize the probability of response in the clinic. Importantly, GSA can also suggest pathways to enrich (too) simplistic turnover models just enough to unlock their predictive capacity. The pragmatic approach we proposed here is being applied within AstraZeneca to PROTAC programs, accelerating the drug discovery pipeline and increasing the chances of success in the clinic.

## Figures and Tables

**Fig. 11:**
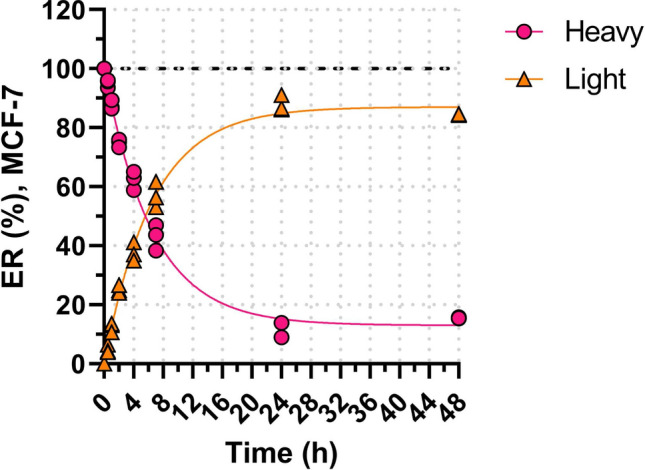
ER$\alpha$ SILAC data in untreated MCF-7 cells

## Appendix A exact solution of bilinear systems

A mathematical method to obtain an exact steady state solution to chemical reaction networks with bilinear rate laws is described in [18] and can be summarized as follows:

1. Linearize the system by treating the bilinear terms (“bilinears”) as dummy variables (“dummies”);
2. Partition the augmented variables (individual variables and dummies) in two sets: *free* variables ($X_F$), which play the role of parameters, and *dependent* variables ($X_D$), which depend on free variables;
3. Obtain the free variables from the dummies definitions and linear conservation laws;
4. Obtain the dependent variables from the free variables calculated at the previous step.

Note that this method cannot be applied to *any* bilinear system. The following two applicability conditions have to be satisfied:

**Requirement 1**: The number of free variables must be “large enough” to contain the states that appear in bilinears. More precisely, the number of variables that appear in bilinear terms ($N_P$) must be less than or equal to the total number of bilinear terms ($N_B$) and conservation laws ($N_C$): $N_P\le N_C+ N_B$.

**Requirement 2**: The choice of free and dependent variables (Step 2) allows the retrieval of free variables from linear relations (Step 3). More precisely, there must exist a *clean set* $\Omega _X$ of $\Delta N= N_P-N_C$ variables which appear in bilinears in such a way that each bilinear term contains at most one of them. If so, dependent variables ($X_D$) will include states that do not appear in bilinears ($X_M$) and a subset of $\Delta N$ bilinears such that each element of the clean set $\Omega _X$ appears once and only once in all the selected bilinears, and free variables ($X_F$) will include states that appear in bilinears and any remaining bilinears not included in $X_D$.

## Appendix B Monovalent degraders

### B.1 Steady state solution

We report the steady state equations derived from the fully mechanistic model (1) for easiness:

B1 $$\begin{aligned} {\left\{ \begin{array}{ll} k_{\text {syn}}- k_{\text {deg}}\cdot \text {T}+ k^{\text {T}}_{\text {off}}\cdot \text {TD}- k^{\text {T}}_{\text {on}}\cdot \text {T}\cdot \text {D}= 0\\ - k^{\text {T}}_{\text {on}}\cdot \text {T}\cdot \text {D}+ (k^{\text {T}}_{\text {off}}+k_{\text {MD}}) \cdot \text {TD}= 0 \\ k^{\text {T}}_{\text {on}}\cdot \text {T}\cdot \text {D}- (k^{\text {T}}_{\text {off}}+k_{\text {MD}}) \cdot \text {TD}= 0 \end{array}\right. } \end{aligned}$$

and define equations and variable sets as in Table 3, with corresponding cardinality.

The first applicability requirement for method [18] is $N_P\le N_C+ N_B$, which is met ($2 \le 1+1$). Although the total number of Equations is $N_E=3$, the linear conservation law introduces constraints among states that reduce the number of *independent* equations to 2 (as shown in Eq. (3)). Therefore we will solve for $N_D=2$ dependent variables and $N_F=N_V-N_D=2$ free variables.

Secondly, we need to find a *clean set* of $\Delta N= N_P-N_C= 1$ variable that appears in bilinears in such a way that each bilinear term contains at most one of them. Because there is only one bilinear term, $\text {T}\cdot \text {D}$, $\text {T}$ or $\text {D}$ are equivalent options. For example, set $\Omega _X= \{\text {T}\}$. The clean set is key to the applicability of the method, as it enables the linearization of the non-linear component of the system.

It is now possible to identify a clean partition of free and dependent augmented variables. Dependent variables ($X_D$) will include states that do not appear in bilinears ($X_M$) and the bilinear term. Free variables ($X_F$) will include states that appear in bilinears ($X_P$) (in this case there are no remaining bilinears since the only one has been included as a dependent variable). In summary:

B2 $$\begin{aligned} \begin{aligned} X_D&= \{ \text {TD}, \text {T}\cdot \text {D}\}\\ X_F&= \{ \text {T}, \text {D}\}. \end{aligned} \end{aligned}$$

With such partition in place, (B1) can be rearranged as a system of 2 independent linear equations in 2 unknown dependent variables $X_D$ parametrized on the free variables $X_F$ (on the right-hand side):

B3 $$\left\{ {\begin{array}{*{20}l} {k_{{{\text{off}}}}^{{\text{T}}} \cdot {\text{TD}} - k_{{{\text{on}}}}^{{\text{T}}} \cdot ({\text{T}} \cdot {\text{D}}) = - k_{{{\text{syn}}}} + k_{{{\text{deg}}}} \cdot {\text{T}}} \hfill \\ {(k_{{{\text{off}}}}^{{\text{T}}} + k_{{{\text{MD}}}} ) \cdot {\text{TD}} - k_{{{\text{on}}}}^{{\text{T}}} \cdot ({\text{T}} \cdot {\text{D}}) = 0} \hfill \\ \end{array} } \right.$$

Solving such linear system provides expressions for the binary complex $\text {TD}$ and the bilinear term as functions of the remaining states $\text {T}$ and $\text {D}$:

B4a $$\begin{aligned} \text {TD}\left( \text {T}\right)&= \dfrac{k_{\text {syn}}}{k_{\text {MD}}} - \dfrac{k_{\text {deg}}}{k_{\text {MD}}}\cdot \text {T} \end{aligned}$$

B4b $$\begin{aligned} \left( \text {T}\cdot \text {D}\right) \left( \text {T}\right)&= \dfrac{k_{\text {syn}}}{k_{\text {MD}}}\dfrac{k^{\text {T}}_{\text {off}}+k_{\text {MD}}}{k^{\text {T}}_{\text {on}}}- \dfrac{k_{\text {deg}}}{k_{\text {MD}}} \dfrac{k^{\text {T}}_{\text {off}}+k_{\text {MD}}}{k^{\text {T}}_{\text {on}}}\cdot \text {T} \end{aligned}$$

Because the bilinear term contains only one variable of the clean set $\Omega _X$ (i.e., it is linear in the clean set), $\text {D}$ can be easily obtained as function of $\text {T}$:

B5 $$\begin{aligned} \text {D}(\text {T}) = \dfrac{k_{\text {syn}}}{k_{\text {MD}}}\dfrac{k^{\text {T}}_{\text {off}}+k_{\text {MD}}}{k^{\text {T}}_{\text {on}}}\cdot \dfrac{1}{\text {T}} - \dfrac{k_{\text {deg}}}{k_{\text {MD}}} \dfrac{k^{\text {T}}_{\text {off}}+k_{\text {MD}}}{k^{\text {T}}_{\text {on}}} \end{aligned}$$

At this point, by plugging in expressions (B4a) and (B5) the conservation law (2) can be expressed by and solved for for $\text {T}$ only:

B6 $$\begin{aligned} \begin{aligned} \text {D}_{tot}(\text {T})&= \text {D}(\text {T}) + \text {TD}(\text {T}) \\&= \dfrac{k_{\text {syn}}}{k_{\text {MD}}} - \dfrac{k_{\text {deg}}}{k_{\text {MD}}}\cdot \text {T}+ \dfrac{k_{\text {syn}}}{k_{\text {MD}}}\dfrac{k^{\text {T}}_{\text {off}}+k_{\text {MD}}}{k^{\text {T}}_{\text {on}}}\cdot \dfrac{1}{\text {T}} \\&\quad - \dfrac{k_{\text {deg}}}{k_{\text {MD}}} \dfrac{k^{\text {T}}_{\text {off}}+k_{\text {MD}}}{k^{\text {T}}_{\text {on}}}= \text {D}_0, \end{aligned} \end{aligned}$$

which is the implicit exact solution (10).

In summary, this method linearizes the original non-linear system (B1) by treating bilinear terms as additional (dummy) variables and by wisely choosing a subset of them as parameters so that the non-linear core of the system is curtailed to the sole conservation laws.

**Table 3:** SERDs - Equations and variable sets with corresponding cardinality

Description	Set	Cardinality
Equations	Eq. (1)	$N_E= 3$
Conservation laws	Eq. (2)	$N_C=1$
States	$X= \{\text {T},\text {D},\text {TD}\}$	$N_E= 3$
Bilinears (dummies)	$X_B=\{\text {T}\cdot \text {D}\}$	$N_B=1$
Augmented variables (states and dummies)	$X\cup X_B$	$N_V= 4$
States that appear in bilinears	$X_P=\{\text {T}, \text {D}\}$	$N_P= 2$
States that do not appear in bilinears	$X_M=\{\text {TD}\}$	$N_E- N_P=1$

### B.2 Exact expressions of total target relative to baseline as a function of free drug

Most *in vitro* assays detect the (fraction of) *total* amount of remaining target $\text {T}_{tot}= \text {T}+ \text {TD}$, whose exact expression as a function of $\text {T}$ can be easily obtained from (B4a):

B7 $$\begin{aligned} \text {T}_{tot}(\text {T}) = \text {TD}(\text {T}) + \text {T}= \dfrac{k_{\text {syn}}}{k_{\text {MD}}} +\left( 1 - \dfrac{k_{\text {deg}}}{k_{\text {MD}}}\right) \cdot \text {T}. \end{aligned}$$

Kinetics parameters such as $k^{\text {T}}_{\text {on}}$ and $k^{\text {T}}_{\text {off}}$ do not appear explicitly, rather implicitly through $\text {T}$. Although finding a formula for $\text {T}$ is impossible — it can only be calculated numerically from (B6) — relationship (B4b) can be used to express $\text {T}$ in terms of $\text {D}$, whereby on and off rates appear explicitly:

B8 $$\begin{aligned} \text {T}= \dfrac{\dfrac{k_{\text {syn}}}{k_{\text {MD}}} \dfrac{k^{\text {T}}_{\text {off}}+k_{\text {MD}}}{k^{\text {T}}_{\text {on}}}}{\left( \text {D}+ \dfrac{k_{\text {deg}}}{k_{\text {MD}}} \dfrac{k^{\text {T}}_{\text {off}}+k_{\text {MD}}}{k^{\text {T}}_{\text {on}}}\right) }. \end{aligned}$$

Plugging (B8) into (B7) and dividing by the baseline $\text {T}_0= k_{\text {syn}}/k_{\text {deg}}$ we obtain the fraction of total remaining target relative to baseline:

B9 $$\begin{aligned} \dfrac{\text {T}_{tot}}{\text {T}_0}(\text {D}) = \dfrac{k_{\text {deg}}}{k_{\text {MD}}} +\left( 1 - \dfrac{k_{\text {deg}}}{k_{\text {MD}}}\right) \cdot \dfrac{\dfrac{k_{\text {deg}}}{k_{\text {MD}}}\dfrac{k^{\text {T}}_{\text {off}}+k_{\text {MD}}}{k^{\text {T}}_{\text {on}}}}{\left( \text {D}+ \dfrac{k_{\text {deg}}}{k_{\text {MD}}} \dfrac{k^{\text {T}}_{\text {off}}+k_{\text {MD}}}{k^{\text {T}}_{\text {on}}}\right) }, \end{aligned}$$

which is Eq. (11).

### B.3 Minimal parametrization of total remaining target relative to baseline

Since the available *in vitro* data is typically in the form of total target as a fraction of baseline, in order to enable parameter estimation we need to express the exact solution (B6) in terms of $\widehat{\text {T}}_{tot}= \text {T}_{tot}/ \text {T}_0$. This task can be performed in three steps:

1. Express (B6) in terms of the normalized free target $\widehat{\text {T}}=\text {T}/\text {T}_0$. By multiplying and dividing each term containing $\text {T}$ in (B6) by the baseline $\text {T}_0=k_{\text {syn}}/k_{\text {deg}}$ (note that this equates to multiplying by 1, which does not change the equation) we obtain:
  B10 $$\begin{aligned}{} & {} - \dfrac{k_{\text {deg}}}{k_{\text {MD}}}\dfrac{k_{\text {syn}}}{k_{\text {deg}}} \cdot \widehat{\text {T}}+ \dfrac{k_{\text {syn}}}{k_{\text {MD}}}\dfrac{k_{\text {deg}}}{k_{\text {syn}}}\dfrac{k^{\text {T}}_{\text {off}}+k_{\text {MD}}}{k^{\text {T}}_{\text {on}}}\cdot \dfrac{1}{\widehat{\text {T}}} \nonumber \\{} & {} \quad + \dfrac{k_{\text {syn}}}{k_{\text {MD}}} - \dfrac{k_{\text {deg}}}{k_{\text {MD}}} \dfrac{k^{\text {T}}_{\text {off}}+k_{\text {MD}}}{k^{\text {T}}_{\text {on}}}= \text {D}_0, \end{aligned}$$
  and recalling the definitions of the surrogate parameters $\alpha$, $\beta$, $\gamma$ (19):
  B11 $$\begin{aligned} - \alpha \cdot \widehat{\text {T}}+ \beta \cdot \gamma \cdot \dfrac{1}{\widehat{\text {T}}} + \alpha - \beta \cdot \gamma = \text {D}_0. \end{aligned}$$
2. Express $\widehat{\text {T}}$ as a function of $\widehat{\text {T}}_{tot}$. By normalizing (B7) we obtain
  B12 $$\begin{aligned} \widehat{\text {T}}_{tot}(\widehat{\text {T}}) = \dfrac{\text {T}_{tot}}{k_{\text {syn}}/k_{\text {deg}}}= \dfrac{k_{\text {deg}}}{k_{\text {MD}}} +\left( 1 - \dfrac{k_{\text {deg}}}{k_{\text {MD}}}\right) \cdot \widehat{\text {T}} \end{aligned}$$
  and inverting for $\widehat{\text {T}}$
  B13 $$\begin{aligned} \widehat{\text {T}}(\widehat{\text {T}}_{tot}) = \dfrac{\widehat{\text {T}}_{tot}- \dfrac{k_{\text {deg}}}{k_{\text {MD}}}}{\left( 1 - \dfrac{k_{\text {deg}}}{k_{\text {MD}}}\right) }= \dfrac{\widehat{\text {T}}_{tot}- \beta }{\left( 1 - \beta \right) } \end{aligned}$$
3. Plug (B13) into (B11). We obtain
  B14 $$\begin{aligned} \begin{aligned}&- \dfrac{\alpha }{1-\beta } \cdot (\widehat{\text {T}}_{tot}-\beta ) + \beta \cdot \gamma \cdot (1-\beta )\cdot \\&\quad \dfrac{1}{\widehat{\text {T}}_{tot}-\beta }+ \alpha -\beta \cdot \gamma = \text {D}_0, \end{aligned} \end{aligned}$$
  which can be expanded as a polynomial function in $\widehat{\text {T}}_{tot}$:
  B15 $$\begin{aligned}{} & {} - \dfrac{\alpha }{1-\beta } \widehat{\text {T}}_{tot}^2 + (\alpha -\beta \gamma + \dfrac{2\alpha \beta }{1-\beta } - \text {D}_0)\widehat{\text {T}}_{tot}\nonumber \\{} & {} \quad -(\alpha -\beta \gamma - \text {D}_0)\beta + \beta \gamma (1-\beta )- \dfrac{\alpha \beta ^2}{1-\beta }=0. \end{aligned}$$

The surrogate parameters $\alpha$, $\beta$, $\gamma$ are still identifiable from (B15), in fact they can be uniquely obtained by estimating

B16 $$\begin{aligned} \begin{aligned} c_1&= \dfrac{\alpha }{1-\beta } \\ c_2&= \alpha -\beta \gamma + \dfrac{2\alpha \beta }{1-\beta } - \text {D}_0\\ c_3&= -(\alpha -\beta \gamma - \text {D}_0)\beta + \beta \gamma (1-\beta )- \dfrac{\alpha \beta ^2}{1-\beta }, \end{aligned} \end{aligned}$$

which are certainly identifiable by construction, and by setting

B17 $$\begin{aligned} \begin{aligned} \beta&= \dfrac{c_2 +c_3 - c_1 +\text {D}_0}{\text {D}_0}\\ \alpha&= c_1(1-\beta ) \\ \gamma&= \dfrac{c_3}{\beta }+c_1-\text {D}_0. \end{aligned} \end{aligned}$$

## Appendix C SILAC modelling

The following mono-exponential model was fitted to heavy- and light-labelled SILAC data in untreated MCF-7 cells (Fig. 11), with the endogenous fractional turnover rate $k_{\text {deg}}$ as a shared parameter:

C18 $$\begin{aligned} \widehat{\text {T}}= (\widehat{\text {T}}_{0} - \widehat{\text {T}}_{\infty }) \cdot e^{-k_{\text {deg}}\cdot t} + \widehat{\text {T}}_{\infty }. \end{aligned}$$

Model estimates are reported in Table 4 with $95\%$ confidence intervals.

**Table 4:** Parameter estimates of model (C18) fitted to the data in Fig. 11

Parameter	Heavy	Light
$k_{\text {deg}}\ (h^{-1})$	$0.1535\ (0.1432-0.1643)$	$0.1535\ (0.1432-0.1643)$
$\widehat{\text {T}}_{0}\ (\%)$	$100.3\ (98.30-102.3)$	$87.02\ (84.74-89.33)$
$\widehat{\text {T}}_{\infty }\ (\%)$	$12.98\ (10.67-15.26)$	$-0.2821\ (-2.28-1.70)$

## Appendix D PROTACs

### D.1 Steady state solution

The steady state of the dynamical system (4) is obtained by setting the time derivatives to 0:

D19 $$\left\{ {\begin{array}{*{20}l} {k_{{{\text{syn}}}} - k_{{{\text{deg}}}} \cdot {\text{T}} + k_{{{\text{off}}}}^{{{\text{PL}}}} \cdot {\text{TPL}} + k_{{{\text{off}}}}^{{\text{T}}} \cdot {\text{PT}} - k_{{{\text{on}}}}^{{\text{T}}} \cdot {\text{T}} \cdot {\text{P}} - k_{{{\text{on}}}}^{{{\text{PL}}}} \cdot {\text{PL}} \cdot {\text{T}} = 0} \hfill \\ {k_{{{\text{off}}}}^{{{\text{PL}}}} \cdot {\text{TPL}} + k_{{{\text{PRO}}}} \cdot {\text{TPL}} - k_{{{\text{off}}}}^{{\text{L}}} \cdot {\text{PL}} + k_{{{\text{on}}}}^{{\text{L}}} \cdot {\text{L}} \cdot {\text{P}} - k_{{{\text{on}}}}^{{{\text{PL}}}} \cdot {\text{PL}} \cdot {\text{T}} = 0} \hfill \\ {k_{{{\text{off}}}}^{{{\text{PT}}}} \cdot {\text{TPL}} - k_{{{\text{MD}}}} \cdot {\text{PT}} - k_{{{\text{off}}}}^{{\text{T}}} \cdot {\text{PT}} + k_{{{\text{on}}}}^{{\text{T}}} \cdot {\text{T}} \cdot {\text{P}} - k_{{{\text{on}}}}^{{{\text{PT}}}} \cdot {\text{PT}} \cdot {\text{L}} = 0} \hfill \\ { - k_{{{\text{PRO}}}} \cdot {\text{TPL}} - k_{{{\text{off}}}}^{{{\text{PL}}}} \cdot {\text{TPL}} - k_{{{\text{off}}}}^{{{\text{PT}}}} \cdot {\text{TPL}} + k_{{{\text{on}}}}^{{{\text{PL}}}} \cdot {\text{PL}} \cdot {\text{T}} + k_{{{\text{on}}}}^{{{\text{PT}}}} \cdot {\text{PT}} \cdot {\text{L}} = 0} \hfill \\ { - k_{{{\text{on}}}}^{{\text{L}}} \cdot {\text{L}} \cdot {\text{P}} - k_{{{\text{on}}}}^{{{\text{PT}}}} \cdot {\text{PT}} \cdot {\text{L}} + k_{{{\text{off}}}}^{{\text{L}}} \cdot {\text{PL}} + k_{{{\text{off}}}}^{{{\text{PT}}}} \cdot {\text{TPL}} = 0} \hfill \\ { - k_{{{\text{on}}}}^{{\text{T}}} \cdot {\text{T}} \cdot {\text{P}} - k_{{{\text{on}}}}^{{\text{L}}} \cdot {\text{L}} \cdot {\text{P}} + k_{{{\text{MD}}}} \cdot {\text{PT}} + k_{{{\text{off}}}}^{{\text{T}}} \cdot {\text{PT}} + k_{{{\text{off}}}}^{{\text{L}}} \cdot {\text{PL}} = 0.} \hfill \\ \end{array} } \right.$$

Let us define equations and variable sets as in Table 5, with corresponding cardinality. The first applicability requirement on dimensionality $N_P\le N_C+ N_B$ is met ($5 \le 2+4$). Although the total number of equations is $N_E=6$, the two linear conservation laws introduce constraints among states that reduce the number of *independent* equations to 4. Therefore we will solve for $N_D=4$ dependent variables ($X_D$) and $N_F=N_V-N_D=6$ free variables ($X_F$).

In order to be able to apply the method, the choice of free and dependent states cannot be fully arbitrary. We need to find a *clean set* of $\Delta N= N_P-N_C= 3$ variables that appear in bilinears in such a way that each bilinear term contains at most one of them. For instance, $\text {P}$, $\text {PT}$ and $\text {PL}$ all appear in the bilinears $X_B$, but each bilinear contains each one of them only once (in other words, $\text {P}$, $\text {PT}$ and $\text {PL}$ are never multiplied by one another). Therefore, $\Omega _X= \{\text {P}, \text {PT}, \text {PL}\}$ is a clean set for system (D19). The clean set is key to the applicability of the method, as it enables the linearization of the non-linear component of the system.

It is now possible to identify a clean partition of free and dependent augmented variables. Dependent variables will include states that do not appear in bilinears ($X_M$) and a subset of $\Delta N=3$ bilinears such that each element of the clean set $\Omega _X$ appears once and only once in all the selected bilinears, for a total of $N_D=4$. For instance, there is one and only one bilinear in the set $X_{B,\Omega _X}= \{ \text {L}\cdot \text {P}, \text {T}\cdot \text {PL}, \text {L}\cdot \text {PT}\}$ that contains each one of $\text {P}$, $\text {PT}$ and $\text {PL}$ (i.e., elements of the clean set $\Omega _X$) once: only $\text {L}\cdot \text {P}$ contains $\text {P}$, only $\text {T}\cdot \text {PL}$ contains $\text {PL}$, and only $\text {L}\cdot \text {PT}$ contains $\text {PT}$.^2^ Free variables will include states that appear in bilinears ($X_P$) and the remaining bilinears ($X_B\setminus X_{B,\Omega _X}$). In summary:

D20 $$\begin{aligned} \begin{aligned} X_D&= X_M\cup X_{B,\Omega _X}= \{ \text {TPL},\text {L}\cdot \text {P}, \text {T}\cdot \text {PL}, \text {L}\cdot \text {PT}\}\\ X_F&= X_P\cup \left( X_B\setminus X_{B,\Omega _X}\right) = \{\text {T}, \text {P}, \text {L}, \text {PT}, \text {PL}, \text {T}\cdot \text {P}\}. \end{aligned} \end{aligned}$$

With such partition in place, system (D19) can be rearranged as a system of 4 independent linear equations in 4 unknown dependent variables $X_D$ parametrized on the free variables $X_F$ (on the right-hand side). Solving such linear system provides expressions for $\text {TPL}$ (i.e., $X_M$) and the bilinears $X_{B,\Omega _X}$ as functions of the remaining states $\text {T}$, $\text {P}$, $\text {L}$, $\text {PT}$, $\text {PL}$ (i.e., $X_P$) and the bilinear $\text {T}\cdot \text {P}$ (i.e., $X_B\setminus X_{B,\Omega _X}$). Thanks to the rational choice of free and dependent variables, the three equations associated with the bilinears $X_{B,\Omega _X}$ in turn make up a *linear* sub-system in the clean variables of $\Omega _X$, parametrized on $\text {T}$ and $\text {L}$ (i.e., $X_P{\setminus } \Omega _X$). As a result, solving this linear sub-system provides expressions for $\text {P}$, $\text {PT}$ and $\text {PL}$ in terms of $\text {T}$ and $\text {L}$, which in turn allows for reformulating the conservation laws as functions solely of the two latter variables. Finally, solving the conservation laws for $\text {T}$ and $\text {L}$ allows the retrieval of all the other states (and dummies).

In summary, this method linearizes the original non-linear system by treating bilinear terms as additional (dummy) variables and by wisely choosing a subset of them as parameters so that the non-linear core of the system is curtailed to the sole conservation laws.

**Table 5:** PROTACs - Equations and variable sets with corresponding cardinality

Description	Set	Cardinality
Equations	Eq. (D19)	$N_E= 6$
Conservation laws	Eq. (7)	$N_C=2$
States	$X= \{\text {T},\text {L},\text {P},\text {PT},\text {PL},\text {TPL}\}$	$N_E= 6$
Bilinears (dummies)	$X_B=\{\text {T}\cdot \text {P}, \text {L}\cdot \text {P}, \text {T}\cdot \text {PL}, \text {L}\cdot \text {PT}\}$	$N_B=4$
Augmented variables (states and dummies)	$X\cup X_B$	$N_V= 10$
States that appear in bilinears	$X_P=\{\text {T}, \text {P}, \text {L}, \text {PT}, \text {PL}\}$	$N_P= 5$
States that do not appear in bilinears	$X_M=\{\text {TPL}\}$	$N_E- N_P=1$

Isolating dependent variables on the left-hand side and free variables on the right-hand side in (D19) yields:

D21 $$\begin{aligned} {\left\{ \begin{array}{ll} k^{\text {PL}}_{\text {off}}\cdot \text {TPL}-k^{\text {PL}}_{\text {on}}\cdot (\text {T}\cdot \text {PL}) = -k_{\text {syn}}+k_{\text {deg}}\cdot \text {T}- k^{\text {T}}_{\text {off}}\cdot \text {PT}+k^{\text {T}}_{\text {on}}\cdot (\text {T}\cdot \text {P}) \\ (k^{\text {PL}}_{\text {off}}+ k_{\text {PRO}}) \cdot \text {TPL}+ k^{\text {L}}_{\text {on}}\cdot (\text {L}\cdot \text {P}) -k^{\text {PL}}_{\text {on}}\cdot (\text {T}\cdot \text {PL}) = k^{\text {L}}_{\text {off}}\\ k^{\text {PT}}_{\text {off}}\cdot \text {TPL}-k^{\text {PT}}_{\text {on}}\cdot (\text {L}\cdot \text {PT}) = (k_{\text {MD}}+k^{\text {T}}_{\text {off}}) \cdot \text {PT}- k^{\text {T}}_{\text {on}}\cdot (\text {T}\cdot \text {P})\\ -(k_{\text {PRO}}+k^{\text {PL}}_{\text {off}}+k^{\text {PT}}_{\text {off}}) \cdot \text {TPL}+ k^{\text {PL}}_{\text {on}}\cdot (\text {T}\cdot \text {PL}) + k^{\text {PT}}_{\text {on}}\cdot (\text {L}\cdot \text {PT})=0 \end{array}\right. } \end{aligned}$$

where the equations for $\text {P}$ and $\text {L}$ have been dropped due to redundancy (they can be retrieved from the remaining states and the conservation laws) and each bilinear term has been highlighted in brackets as a whole dummy variable. System (D21) can be written in matrix form as

D22 $$\begin{aligned}{} & {} \left[ \begin{array}{llll} k^{\text {PL}}_{\text {off}}&{} 0 &{} -k^{\text {PL}}_{\text {on}}&{} 0 \\ k^{\text {PL}}_{\text {off}}+ k_{\text {PRO}}&{} k^{\text {L}}_{\text {on}}&{} -k^{\text {PL}}_{\text {on}}&{} 0 \\ k^{\text {PT}}_{\text {off}}&{} 0 &{} 0 &{} -k^{\text {PT}}_{\text {on}}\\ -(k_{\text {PRO}}+k^{\text {PL}}_{\text {off}}+k^{\text {PT}}_{\text {off}}) &{} 0 &{} k^{\text {PL}}_{\text {on}}&{} k^{\text {PT}}_{\text {on}}\end{array} \right] \begin{bmatrix} \text {TPL}\\ \text {L}\cdot \text {P}\\ \text {T}\cdot \text {PL}\\ \text {L}\cdot \text {PT}\end{bmatrix}\nonumber \\{} & {} \quad = - \left[ \begin{array}{lllllll} k_{\text {syn}}&{} -k_{\text {deg}}&{} 0 &{} k^{\text {T}}_{\text {off}}&{} 0 &{} 0 &{} -k^{\text {T}}_{\text {on}}\\ 0 &{} 0 &{} -k^{\text {L}}_{\text {off}}&{} 0 &{} 0 &{} 0 &{} 0 \\ 0 &{} 0 &{} 0 &{} -(k_{\text {MD}}+k^{\text {T}}_{\text {off}}) &{} 0 &{} 0 &{} k^{\text {T}}_{\text {on}}\\ 0 &{} 0 &{} 0 &{} 0 &{} 0 &{} 0 &{} 0 \end{array} \right] \begin{bmatrix} 1 \\ \text {T}\\ \text {PL}\\ \text {PT}\\ \text {L}\\ \text {P}\\ \text {T}\cdot \text {P}\\ \end{bmatrix} \end{aligned}$$

with solution (see Appendix D.2 for coefficients definitions)

D23a $$\begin{aligned} \text {TPL}&= t_{00} + t_{10} \cdot \text {T}+t_{\star }\cdot \text {PT} \end{aligned}$$

D23b $$\begin{aligned} \text {L}\cdot \text {P}&= c_{11}\text {PL}+ c_{12}\text {PT}- c_{13} \cdot \text {P}\cdot \text {T} \end{aligned}$$

D23c $$\begin{aligned} \text {T}\cdot \text {PL}&= c_{20} - c_{21} \text {T}+ c_{22} \text {PT}- c_{23} \cdot \text {P}\cdot \text {T}\end{aligned}$$

D23d $$\begin{aligned} \text {L}\cdot \text {PT}&= c_{30} - c_{31} \text {T}- c_{32} \text {PT}+ c_{33} \cdot \text {P}\cdot \text {T}. \end{aligned}$$

Eqs. (D23b)-(D23d) form a linear system in the clean set $\Omega _X=\{\text {P}, \text {PT}, \text {PL}\}$, parametrized on $\text {T}$ and $\text {L}$, which can be recast in matrix form as

D24 $$\begin{aligned} \begin{bmatrix} -c_{13}\cdot \text {T}- \text {L}&{} c_{11} &{} c_{12} \\ c_{23} \cdot \text {T}&{} \text {T}&{} -c_{22} \\ c_{33} \cdot \text {T}&{} 0 &{}-c_{32} - \text {L}\end{bmatrix} \begin{bmatrix} \text {P}\\ \text {PL}\\ \text {PT}\end{bmatrix} = \begin{bmatrix} 0 \\ c_{20} - c_{21} \text {T}\\ -c_{30} + c_{31} \text {T}\\ \end{bmatrix}, \end{aligned}$$

with solution

D25a $$\begin{aligned} \text {P}&= \dfrac{ -p_{20}\text {T}^2 - p_{11}\text {L}\cdot \text {T}+ p_{10}\text {T}+ p_{01}\text {L}+ p_{00} }{\text {T}(\text {L}^2 + \delta _{11}\text {L}\cdot \text {T}+ \delta _{01} \text {L}+\delta _{10}\text {T}+ \delta _{00})} \end{aligned}$$

D25b $$\begin{aligned} \text {PL}&= \dfrac{ b_{L,02}\text {L}^2 - b_{L,12}\text {L}^2\cdot \text {T}- b_{L,21}\text {L}\cdot \text {T}^2 + b_{L,20}\text {T}^2 + b_{L,01}\text {L}- b_{L,10}\text {T}+ b_{L,11}\text {L}\cdot \text {T}}{\text {T}(\text {L}^2 + \delta _{11}\text {L}\cdot \text {T}+ \delta _{01} \text {L}+\delta _{10}\text {T}+ \delta _{00})}\end{aligned}$$

D25c $$\begin{aligned} \text {PT}&= \dfrac{\text {T}(- b_{T,20}\text {T}^2 + b_{T,01}\text {L}- b_{T,11}\text {L}\cdot \text {T}+ b_{T,10}\text {T}+ b_{T,00})}{\text {T}(\text {L}^2 + \delta _{11}\text {L}\cdot \text {T}+ \delta _{01} \text {L}+\delta _{10}\text {T}+ \delta _{00})} \end{aligned}$$

(see Appendix D.2 for coefficients definitions). Finally, plugging expressions (D23a) and (D25a)-(D25c) in the conservation laws (7) provides a system of two non-linear equations in two unknowns:

D26 $$\left\{ {\begin{array}{*{20}l} {{\text{L}}_{{tot}} ({\text{T}},{\text{L}})\, = \,{\text{L}}\, + \,{\text{PL}}({\text{T}},{\text{L}})\, + \,{\text{TPL}}({\text{T}},{\text{L}})\, = \,{\text{L}}_{0} } \hfill \\ {{\text{P}}_{{tot}} ({\text{T}},{\text{L}})\, = \,{\text{P}}({\text{T}},{\text{L}})\, + \,{\text{PT}}({\text{T}},{\text{L}})\, + \,{\text{PL}}({\text{T}},{\text{L}})\, + \,{\text{TPL}}({\text{T}},{\text{L}})\, = \,{\text{P}}_{0} ,} \hfill \\ \end{array} } \right.$$

from which $\text {T}$ and $\text {L}$ can be numerically calculated for any given concentration $\text {P}_0$ and ligase baseline $\text {L}_0$.

### D.2 Steady state solution coefficients

The rationale for coefficients notation is displayed in Table 6. The subscripts in each coefficients are determined as follows:

- $c_{ij}$ is the coefficient in row *i*, column *j* of the clean set system in matrix form (D24);
- $t_{ij}$, $p_{ij}$, $b_{T,ij}$, $b_{L,ij}$, $\delta _{ij}$ multiply the monomial term $\text {T}^i \cdot \text {L}^j$ in the corresponding formula as in Table 6;
- $t_{\star }$ multiplies the $\text {PT}$ binary complex in (D23a) (and is the only coefficient that does not multiply a monomial term in two variables of the form $\text {T}^i \cdot \text {L}^j$).

**Table 6:** Coefficient notation key for all (set of) variables

Coefficient notation	Variable (set)	Equation reference
$$t_{**}$$	$${\underline{t}}$$ernary complex ($$\text {TPL}$$)	(D23a)
$$c_{**}$$	$${\underline{c}}$$lean set system ($$\text {P}$$, $$\text {PT}$$, $$\text {PL}$$)	(D23b)-(D23d), (D24)
$$p_{**}$$	$${\underline{P}}$$ROTAC ($$\text {P}$$)	(D25a)
$$b_{T,**}$$	PROTAC-$${\underline{T}}$$arget $${\underline{b}}$$inary complex ($$\text {PT}$$)	(D25c)
$$b_{L,**}$$	PROTAC-$${\underline{L}}$$igase $${\underline{b}}$$inary complex ($$\text {PL}$$)	(D25b)
$$\delta _{**}$$	$${\underline{d}}$$enominator of the clean set variables (and $${\underline{d}}$$eterminant of the clean set system (D24))	(D25a)-(D25c)

Eqs. (D23a)-(D23d), (D24)

$$\begin{aligned} \begin{aligned} c_{11}&= \dfrac{k^{\text {L}}_{\text {off}}}{k^{\text {L}}_{\text {on}}}\\ c_{12}&= \dfrac{k^{\text {T}}_{\text {off}}}{k^{\text {L}}_{\text {on}}}+\dfrac{k_{\text {MD}}}{k^{\text {L}}_{\text {on}}}\\ c_{13}&= \dfrac{k^{\text {T}}_{\text {on}}}{k^{\text {L}}_{\text {on}}} \\ c_{20}&= \dfrac{k^{\text {PL}}_{\text {off}}}{k^{\text {PL}}_{\text {on}}}\cdot \dfrac{k_{\text {syn}}}{k_{\text {PRO}}} + \dfrac{k_{\text {syn}}}{k^{\text {PL}}_{\text {on}}} \\ c_{21}&= \dfrac{k_{\text {deg}}}{k^{\text {PL}}_{\text {on}}}+ \dfrac{k^{\text {PL}}_{\text {off}}}{k^{\text {PL}}_{\text {on}}}\cdot \dfrac{k_{\text {deg}}}{k_{\text {PRO}}} \\ c_{22}&= \dfrac{k^{\text {T}}_{\text {off}}}{k^{\text {PL}}_{\text {on}}} - \dfrac{k^{\text {PL}}_{\text {off}}}{k^{\text {PL}}_{\text {on}}}\cdot \dfrac{k_{\text {MD}}}{k_{\text {PRO}}} \\ c_{23}&= \dfrac{k^{\text {T}}_{\text {on}}}{k^{\text {PL}}_{\text {on}}} \end{aligned}\qquad \begin{aligned} c_{30}&= \dfrac{k^{\text {PT}}_{\text {off}}}{k^{\text {PT}}_{\text {on}}}\cdot \dfrac{k_{\text {syn}}}{k_{\text {PRO}}} \\ c_{31}&= \dfrac{k^{\text {PT}}_{\text {off}}}{k^{\text {PT}}_{\text {on}}} \cdot \dfrac{k_{\text {deg}}}{k_{\text {PRO}}} \\ c_{32}&= \dfrac{k^{\text {T}}_{\text {off}}}{k^{\text {PT}}_{\text {on}}} + \dfrac{k^{\text {PT}}_{\text {off}}}{k^{\text {PT}}_{\text {on}}}\cdot \dfrac{k_{\text {MD}}}{k_{\text {PRO}}} + \dfrac{k_{\text {MD}}}{k^{\text {PT}}_{\text {on}}} \\ c_{33}&= \dfrac{k^{\text {T}}_{\text {on}}}{k^{\text {PT}}_{\text {on}}}\\ t_{00}&= \dfrac{k_{\text {syn}}}{k_{\text {PRO}}} \\ t_{10}&= - \dfrac{k_{\text {deg}}}{k_{\text {PRO}}} \\ t_{\star }&= - \dfrac{k_{\text {MD}}}{k_{\text {PRO}}} \end{aligned} \end{aligned}$$

Eqs. (D25a)-(D25c)

$$\begin{aligned} \begin{aligned} \delta _{11}&= c_{13} \\ \delta _{01}&= c_{11}c_{23} + c_{32} \end{aligned} \qquad \begin{aligned} \delta _{10}&= -c_{12}c_{33} + c_{13}c_{32} \\ \delta _{00}&= - c_{11}c_{22}c_{33} + c_{11}c_{23}c_{32} \end{aligned} \end{aligned}$$

Eq. (D25a)

$$\begin{aligned} \begin{aligned} p_{20}&= -c_{12}c_{31} \\ p_{11}&=- c_{11}c_{21} \\ p_{10}&= c_{12}c_{30} - c_{11}c_{21}c_{32} - c_{11}c_{22}c_{31} \end{aligned} \qquad \begin{aligned} p_{01}&= c_{11}c_{20} \\ p_{00}&= c_{11}c_{20}c_{32} + c_{11}c_{22}c_{30}\\ \end{aligned} \end{aligned}$$

Eq. (D25b)

$$\begin{aligned} \begin{aligned} b_{L,02} =&c_{20} \\ b_{L,12} =&- c_{21} \\ b_{L,21} =&- c_{13}c_{21} \\ b_{L,11} =&c_{13}c_{20} - c_{21}c_{32} - c_{22}c_{31} \end{aligned}\qquad \begin{aligned} b_{L,20} =&c_{12}c_{21}c_{33} + c_{12}c_{23}c_{31} \\&- c_{13}c_{21}c_{32} - c_{13}c_{22}c_{31} \\ b_{L,01} =&c_{20}c_{32} + c_{22}c_{30} \\ b_{L,10} =&- (c_{20}c_{33} +c_{23}c_{30})c_{12} \\&+ (c_{20}c_{32} + c_{22}c_{30})c_{13} \end{aligned} \end{aligned}$$

Eq. (D25c)

$$\begin{aligned} \begin{aligned} b_{T,20}&= - c_{13}c_{31} \\ b_{T,01}&= c_{30} \\ b_{T,11}&= - c_{31} \end{aligned} \qquad \begin{aligned} b_{T,10}&= c_{13}c_{30} - c_{11}c_{21}c_{33} - c_{11}c_{23}c_{31} \\ b_{T,00}&= c_{11}c_{20}c_{33} + c_{11}c_{23}c_{30} \end{aligned} \end{aligned}$$

### D.3 Mechanistic parameters retrieval

Assume endogenous degradation rate $k_{\text {deg}}$ and the PROTAC-target binding affinity $k^{\text {T}}_{\text {on}}/k^{\text {T}}_{\text {off}}$ are known form measurements, and that the 9 coefficients $t_{00}$, $t_{10}$, $t_{\star }$, $c_{11}$, $c_{13}$, $c_{20}$, $c_{23}$, $c_{30}$, $c_{33}$ are estimated. Then, synthesis and degradation rates are obtained as:

$$\begin{aligned} \begin{aligned} k_{\text {syn}}&= - k_{\text {deg}}\dfrac{t_{00}}{t_{10}} \\ k_{\text {MD}}&= k_{\text {deg}}\dfrac{t_{10}}{t_{\star }} \\ k_{\text {PRO}}&= \dfrac{k_{\text {syn}}}{t_{00}} \end{aligned} \end{aligned}$$

From the definitions of $c_{11}$ and $c_{30}$, and given the ratio $k^{\text {T}}_{\text {on}}/k^{\text {T}}_{\text {off}}$, it follows

$$\begin{aligned} \begin{aligned} \dfrac{k^{\text {PT}}_{\text {off}}}{k^{\text {PT}}_{\text {on}}}&= c_{30}\dfrac{k_{\text {PRO}}}{k_{\text {syn}}} \\ \alpha&= \dfrac{c_{30}}{c_{11}}\dfrac{k_{\text {PRO}}}{k_{\text {syn}}} \\ \dfrac{k^{\text {PL}}_{\text {off}}}{k^{\text {PL}}_{\text {on}}}&= \alpha \dfrac{k^{\text {T}}_{\text {off}}}{k^{\text {T}}_{\text {on}}} \end{aligned} \end{aligned}$$

and finally

$$\begin{aligned} \begin{aligned} k^{\text {PL}}_{\text {on}}&= \dfrac{k_{\text {syn}}}{c_{20} - \dfrac{k^{\text {PL}}_{\text {off}}}{k^{\text {PL}}_{\text {on}}} \cdot \dfrac{k_{\text {syn}}}{k_{\text {PRO}}}} \\ k^{\text {T}}_{\text {on}}&= k^{\text {PL}}_{\text {on}}\cdot c_{23} \\ k^{\text {L}}_{\text {on}}&= \dfrac{k^{\text {T}}_{\text {on}}}{c_{13}} \\ k^{\text {PT}}_{\text {on}}&= \dfrac{k^{\text {T}}_{\text {on}}}{c_{33}} \\ k^{\text {L}}_{\text {off}}&= k^{\text {L}}_{\text {on}}\cdot c_{11} \\ k^{\text {PT}}_{\text {off}}&= \alpha \dfrac{k^{\text {L}}_{\text {off}}}{k^{\text {L}}_{\text {on}}} k^{\text {PT}}_{\text {on}}\\ k^{\text {PL}}_{\text {off}}&= \alpha \dfrac{k^{\text {T}}_{\text {off}}}{k^{\text {T}}_{\text {on}}} k^{\text {PL}}_{\text {on}}\\ k^{\text {T}}_{\text {off}}&= \dfrac{k^{\text {PL}}_{\text {off}}}{k^{\text {PL}}_{\text {on}}}\dfrac{k^{\text {L}}_{\text {off}}}{k^{\text {L}}_{\text {on}}}\dfrac{k^{\text {PT}}_{\text {on}}}{k^{\text {PT}}_{\text {off}}} k^{\text {T}}_{\text {on}}\end{aligned} \end{aligned}$$

## Footnotes

1 Due to non-linearity, in principle it is not guaranteed that inverting the conservation laws (14) for $\text {T}$ and $\text {L}$ provides a unique solution. Nevertheless, it has been verified numerically that this is the case for the biological and pharmacological systems of interest.
